# Listening to children with lower limb loss: Rationale, design, and protocol for delivery of a novel globally applicable research toolkit—Prosthetic user needs, quality of life, pain, and physical function

**DOI:** 10.1371/journal.pone.0310848

**Published:** 2024-10-31

**Authors:** Caitlin E. Edgar, Claudia Ghidini, Thearith Heang, Clement D. Favier, Craig H. Gerrand, Sherron H. Furtado, Emily R. Mayhew, Sisary Kheng, Ghassan S. Abu-Sittah, Anthony M. J. Bull

**Affiliations:** 1 Department of Bioengineering, The Centre for Paediatric Blast Injury Research, Imperial College London, London, United Kingdom; 2 Exceed Worldwide and The Department of Prosthetics and Orthotics, Phnom Penh, Cambodia; 3 Department of Bioengineering, Imperial College London, London, United Kingdom; 4 Department of Orthopaedic Oncology, Royal National Orthopaedic Hospital, Brockley Hill, Stanmore, United Kingdom; 5 Global Health Institute, Conflict Medicine Program, American University of Beirut, Beirut, Lebanon; Iran University of Medical Sciences, ISLAMIC REPUBLIC OF IRAN

## Abstract

**Introduction:**

Rehabilitation after childhood lower limb loss is complex and dependent on multiple stakeholders and environmental factors. While research with adults underscores the importance of involving prosthetic limb users and caregivers in discussions to drive innovation, children are often excluded or not effectively engaged. This protocol lays out the development and implementation protocol for an internationally applicable research toolkit which has been designed and evaluated around the essential presence of the child. Research domains span their unique prosthetic needs, quality of life, pain, and mobility.

**Methods and analysis:**

Cohorts of children in contrasting environments were identified (Cambodia, Gaza Strip, and the UK) to provide a comprehensive global understanding of the child with lower limb loss. A literature review revealed a lack of appropriate tools for identifying paediatric prosthetic user requirements leading to the development of novel interview guides for each key stakeholder: child, caregiver, and prosthetist. The child’s guide centred around enjoyment and engagement using card games and activities. A panel of experts in paediatric limb loss and mental health rigorously reviewed the guides. Guides were integrated with existing validated measures for quality of life, pain, and mobility to form a comprehensive toolkit. The toolkit was successfully piloted with 5 children, their families, and 2 prosthetists.

This protocol lays out the toolkit rationale and implementation plan (Jan 2023 to Dec 2025). This work offers the opportunity for this cohort to enjoyably engage with research that seeks to radically improve prospects for all children living with limb loss. The outlined best practices ensure ethical considerations when working with vulnerable cohorts.

**Ethics and dissemination:**

This study is approved to cover implementation at all geographical locations as well as the researcher institutions. Results will be disseminated through national and international conferences, as well as through manuscripts in leading peer-reviewed journals.

## Introduction

Children with lower limb loss form a unique and significant global cohort who face a complex and challenging rehabilitation process. This challenge is heightened in austere environments such as low- and middle-income countries (LMICs) or conflict/post-conflict zones. An estimated 64% of people living with amputation (65 million in 2020) are in LMICs, with children bearing most of the burden of disability [1]. Also, in 2017, 420 million children were living in conflict affected areas [2–4], where traumatic injuries such as lower limb loss are common [5]. Resource and innovation are primarily focussed on adult lower limb prosthetics and scaled down versions do not consider the child’s unique needs [6]. A recent report from UNICEF ‘found no research or development on products for LMIC/emergencies that specifically considered the children’s needs’ [7].

Set in the broader medical-humanitarian context, this protocol aligns with *The Lancet’*s “Goals at 200” programme which recognises the importance of the needs of children and adolescents: “children need to be immediately prioritised in health and social policies–children and young people deserve attention in their own right and not only because they are an indispensable foundation for a sustainable future.” [8] The specific user needs for this global cohort are not known and will only be addressed if the child and their caregivers are given a voice to define their needs and call for actionable change. This will then enable novel design and clinical care solutions to be identified [2]. The aim of this protocol is to engage with the appropriate stakeholders to develop and implement an internationally applicable research toolkit for children.

To succeed, it must be recognised that children with lower limb loss differ significantly from adults due to physical and mental growth and development [6,9]. Children must be included in the conversation as health research based primarily on adult reports “risk misrepresenting children with disabilities and their needs” [10]. Previous work in this field has succeeded in involving adults who lost their limbs as children into the conversation but few have utilised appropriate methods to involve children themselves [11]. By following existing guidelines on the effective inclusion of children in healthcare related research, these individuals can provide a unique perspective in a safe, fun, and engaging environment [12].

However, the child is just one stakeholder in the prosthetic user chain. Clinicians can provide specific insights into prosthetic component supply, rehabilitation, and prosthetic limb fitting [13–17], while parents can provide a holistic view of their child’s life from self-care to community living and the overall burden of injury. Few published studies for children with lower limb loss have included the full user chain: child, parent, and clinician [18–22]. Chami et al. effectively incorporated the opinions of all 3 users [23]. However as with other previous works for both adults and children the focus has been on overarching challenges treating the prosthetic leg as one full system [24–26] or focussing primarily on specific areas such as cosmesis or participation [22]. As such, designers of individual prosthetic componentry have little detail on understanding the individual challenges a child faces with specific components. The result is slow and ineffective design innovation. Additionally, many studies focus on only one level of lower limb loss, e.g., transtibial, and are therefore unable to identify similarities and differences in user needs between levels. Delivery of this toolkit to a cohort with differing amputation levels will provide comprehensive understanding to the field.

Prosthetic provision is just one aspect of rehabilitation, with physical health, pain, quality of life (QoL) and functional movement forming other key domains [27]. However, there is a scarcity of literature that examines these domains, resulting in the impact of childhood limb loss on these crucial metrics largely unknown [28]. No studies have investigated QoL in differing cultural and societal environments where the cause of amputation, available prosthetic componentry, and rehabilitation protocols likely differ [29]. In fact, all research with children with limb loss to date targets single cohorts in discrete locations or omits large sectors of global cultures. To truly form a holistic understanding of children with lower limb loss it is essential to include as wide a variety of participants as possible into similar, impactful studies.

This protocol lays out the rationale and implementation plan for an internationally applicable research toolkit for children with limb loss in three countries (Cambodia, Gaza, and the UK). The toolkit will contain outcome measures spanning the four domains of childhood limb loss rehabilitation: QoL, pain, functional ability, and prosthetic componentry provision.

## Methods and analysis

### Study overview

The study was delivered in five stages:

1. A literature review conducted prior to 2023 identified which domains of rehabilitation and prosthetic provision should be included and whether existing outcome measures appropriately covered these domains.
2. An interactive interview guide was developed for children with lower limb loss, and iteratively validated with a panel of experts and multiple pilot tests. Additional interview guides for parent/guardians and one of the clinical caregivers, a prosthetist, were created using the same method. The interviews were translated to the local language.
3. Existing validated outcome measures to cover all remaining identified key domains were incorporated.
4. The protocol was framed in the appropriate context by recognising the key requirements for involving children in research and following guidelines for the delivery of research in low- and middle-income countries [12,30].
5. Deployment started in one study location in January 2023 and is on-going.

The target populations for this study are children who have undergone major lower limb amputation due to any aetiology, their parent/legal guardian, and their primary clinical prosthetic caregiver i.e., prosthetist. Major amputation is defined as any level proximal to the ankle joint. All aetiologies of amputation, such as congenital vs acquired, are included to provide a breadth of perspectives. Differences in results according to aetiology may be analysed depending on recruited participant demographics, however it is not a primary objective of this work. Children aged 6-18yrs, male or female, will be recruited to represent the full spectrum of changes throughout growth and development. Children younger than 6yrs are excluded due to considerations on their ability to participate effectively. Age is not a direct measure of cognitive competency, however below 6yrs children can have minimal understanding on healthcare terminology and may misinterpret questions [31,32]. Bevans et al. suggest parent/caregiver reports may be necessary for such a young age [31]. Parents/legal guardians must be present and legally responsible for the child in question and thus able to provide written consent for them to participate in the study. The full inclusion/exclusion criteria is summarised in Table 1.

**Table 1:** Inclusion and exclusion criteria of the research study.

User	Inclusion Criteria	Exclusion Criteria
**Child**	Male or Female	Older than 18 years
	Aged 6-18yrs.	Younger than 5 years
	At least one major lower limb amputation	Any recognized disability other than limb loss (cognitive and/or physical)
	Has first accessed prosthetic clinic services at least 6 months prior	Unable to attend with a parent/guardian who may provide consent
	Healthy and active	Accessed prosthetic clinic services for the first time less than 6 months ago.
	Can attend data collection with parent/guardian aged 18yrs or older	
**Parent/Caregiver**	Male or Female	Younger than 18yrs
	Over 18yrs old	Not parent/legal guardian of paediatric participant
	Parent or Legal Guardian of paediatric participant	Any recognised cognitive impairment
	No cognitive impairment	
**Prosthetist**	Male of Female	Younger than 18yrs
	Over 18yrs old	Never treated paediatric participants who satisfy the above inclusion criteria
	Treating/have recently treated paediatric participants who satisfy the above inclusion criteria	Any recognized cognitive impairment.
	No cognitive impairment	

The toolkit will be delivered in multiple locations to provide worldwide generalisability. Cambodia, Gaza strip and the UK were a convenience sample selected as the locations representing three distinct demographics (Table 2).

**Table 2:** Toolkit deployment locations representing a convenience sample with maximum generalisability.

Location	Cambodia	Gaza Strip *	United Kingdom
**World Bank Country Classification [33]**	Lower-middle income	Upper-middle income	High income
**Recent Conflict History**	Post-Conflict	Conflict	None
**Climate [34]**	Tropical	Dry	Temperate
**Culture**	Austroasiatic language; Buddhist; Eastern	Semitic Language, Islam, Arabic	Indo-European language; Secular (Post-Christian), Western
**Clinical Providers**	Non-Governmental Organisations	Non-Governmental Organisations, Ministry of Health	National Health Service
**Prosthetic Technology**	Polypropylene (International Committee of the Red Cross Designs); Donated modular componentry)	Donated modular componentry	Modular/State of the art

* Information on prosthetic provision refers to known information prior October 7^th^, 2023. Current status is unknown, but prosthetic provision is most likely completely disrupted.

Recruitment of participants started in January 2023 across 3 centres in Cambodia affiliated with the Department of Prosthetics and Orthotics (DPO) Cambodia and Exceed Worldwide in Phnom Penh, Kampong Chhnang Physical, and Kampong Som [35]. Recruitment started in March 2024 in the UK in the National Health Service (NHS) Royal National Orthopaedic Hospital (RNOH) in Stanmore. Recruitment was planned in Gaza at the Hamad Rehabilitation and Prosthetic Hospital this is being reconsidered in the light of the current conflict and another representative location may be chosen. These prosthetic centres provide care for children with limb loss and have a database through which potential participants can be contacted.

## Toolkit development

### Literature review

The biopsychosocial model from the World Health Organisation (WHO) on the International Classification of Functioning, Disability, and Health: Children and Youth Version (ICF-CY) was analysed and provided essential domains for consideration in successful rehabilitation for children with limb loss [27]. These domains cover: QoL including Community Integration, Social Support, and Psychological Impact; Pain and Physical Health; and Functional mobility. These domains have been used by others [36], however, they do not cover user requirements for prosthetic design. Therefore, these factors have now been combined into 4 overall domains: ‘Quality of Life’; ‘Pain and Physical Health’; ‘Functional Mobility’ and ‘Prosthetic Design User Needs’. The literature review discusses the inclusion of existing outcome measures for each domain according to the criteria below:

1. manageable duration/length for completion;
2. appropriate language complexity and wording for each age group; and
3. enjoyment and engagement.

These criteria ensure that the child user can participate enjoyably and effectively [37].

#### Domain 1: Quality of life (community integration; social support; psychological impact)

Questionnaires to assess QoL across all domains (school, home, physical health) for healthy children and those with musculoskeletal disorders were reviewed. Eight questionnaires were found [24,38–44]. Some questionnaires were only appropriate for specific age groups, were very long in duration, or required the use of specific expensive equipment such as a laptop/computer which could not be assumed in some austere environments [24,40,43,44]. Two questionnaires for children with and without disabilities are comprehensive in assessing participation in daily activities [38,39], but do not satisfy the duration design requirement and have not been extensively used.

The Pediatric Quality of Life (PedsQL) 4.0 Generic Core Scales was selected. It is a modular instrument designed to measure QoL in children and adolescents aged 5–18 years and is available in Khmer, Arabic and English thus ready for deployment in the three locations. The administration time satisfies the design requirement (<4 min) [45]. It is sensitive to cognitive development, and it integrates both child self-report and parent proxy-report [46,47]. PedsQL 4.0 distinguishes between healthy children and paediatric patients with acute or chronic health conditions [41,47] and has been used for children with other musculoskeletal disorders which allows for comparisons to be made [48]. The questionnaire measures the core physical, mental, and social health dimensions, as well as school functioning which are the most important health related QoL domains for children with limb deformities [36]. A more recently developed questionnaire specific to children with limb deformity, LIMB-Q; [42], is currently being validated and may be appropriate for future use.

#### Domain 2: Pain and physical health

**Fig 1:**
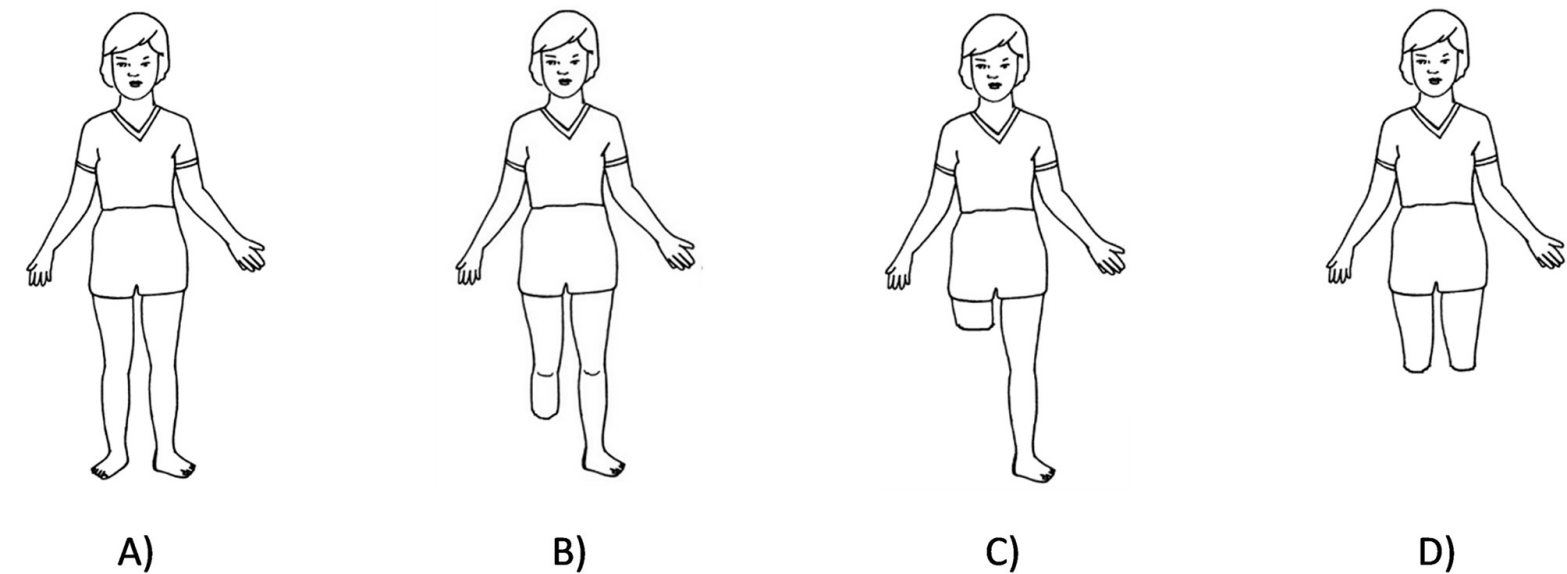
PedsQL pain drawing templates. A) Original image in the PedsQL–PPQ. Modified image to represent B) Transtibial, C) Transfemoral, D) Bilateral amputation. Additional appropriate images for hip and knee disarticulation type of amputations were made.

Residual limb pain, phantom limb pain and secondary physical conditions are often associated with amputation [49,50]. There is a scarcity of research in this area for the paediatric cohort, and it is key to understand prevalence of residual limb conditions and pain to improve their prosthetic fit and rehabilitation [51]. Ten tools were considered for inclusion [25,46,52–56]. One was removed as it was designed only for adults [25]. Others utilised individual techniques which were available as one whole validated package in the form of the PedsQL 4.0—Paediatric Pain Questionnaire (PPQ) [47]. This tool combines two methods to assess pain effectively in children: rating on a child-friendly visual scale and colouring [57]. The child is asked to rate their level of pain and explain where it is in the body by colouring the affected areas. It covers the same age range as the Generic Core Scales and is self- and parent- proxy reported. It takes approximately 10 minutes to administer (author-provided information). Permission from the author of the PedsQL Inventory was sought and granted to modify the body sketches used in the questionnaire and make sure they were representative of these cohorts (any level of lower-limb amputation; Fig 1). Little research has been done on how best to discuss phantom limb pain with children and questions on this are not included in the PedsQL PPQ. It was decided not to add questions on this topic due to the lack of research and potential to trigger upsetting emotions.

PedsQL PPQ can be combined with the Socket Comfort Score (SCS) to increase the specificity of data collected in this domain to children with lower limb loss. The SCS assesses satisfaction with prosthetic fit which has been shown to be a significant issue for pain [52,58]. SCS is a validated, simple numerical scale which records the socket comfort of a prosthetic limb, and it is strongly linked to clinical evidence of poor fitting [52]. Administration lasts approximately 2 minutes [52]. Although it has only been used with adult amputees, the simplicity of the administration and level of understanding required are appropriate for children.

While determining the pain levels felt by the child are crucial, it is also essential to understand the potential causal factors. This knowledge can be provided by a professional clinical evaluation of the residual limb. Potential health conditions for children are bony overgrowth, blistering and ulceration [59–62]. Clinical forms used at multiple prosthetic rehabilitation centres across Cambodia were used as inspiration. The information was combined to create a clinical evaluation form which would both inform the research study and still ensure necessary data for a routine clinical appointment were acquired. As such the sheet included details on prosthetic componentry prescribed as well as anthropometric measures necessary for casting. Administration would last approximately 10 minutes.

In total thirteen tools were considered for inclusion and three chosen: PedsQL PPQ, SCS and a clinical evaluation form. The total administration time for the child and parent was 12 and 10 minutes, respectively. For the clinician, it was 10 minutes.

#### Domain 3: Physical functional capacity

Thirteen tools were found that can assess physical functional capacity in children with physical disabilities [24,40,63–75]. Most were developed for children with cerebral palsy and were not directly applicable [40,71,72]. Other tools were only designed and validated for adults (Prosthetic Limb Users Survey of Mobility, PLUS-M; Special Interest Group in Amputee Medicine, SIGAM; Orthotics and Prosthetics Users Survey, OPUS; The Lower Extremity Functional Scale, LEFS) [24,63,64,74]. These either used complex language, incorporated questions irrelevant to children, were not generalisable to all settings (e.g., ‘Using escalators’), and/or are self-reported thus increasing self-bias in this cohort. Outside scorers remove this bias.

The Pediatric Outcomes Data Collection Instrument (PODCI) was identified as potentially suitable. It is a valid and reliable instrument for assessing functional outcomes in paediatric orthopaedics (e.g., acquired scoliosis, cerebral palsy, congenital leg-length discrepancy) [65,66]. However, the instrument can only be delivered to children aged 11-18yrs as caregivers must complete the form for younger children. It is long (83 questions) and self-reported. The validated Child Amputee Prosthetics Project-Functional Status Inventory (CAPP-FSI) could not be used as it is a proxy-report questionnaire for this cohort focussing primarily on paediatric upper-limb amputees with few lower limb questions [76–78].

The Amputee Mobility Predictor (AMP) [79] was selected as the foundation for the questionnaire. It has been successfully used with paediatric lower-limb amputees previously [68]. It is interactive and the participant is observed performing the activity, removing self-bias associated with self-reporting [63]. Additionally, the response options provide a grading system that allows for an in depth understanding [64].

Nevertheless, the AMP omits important cultural functional aspects and some activities, with an emphasis on basic activities (e.g., 4 different tests for standing). Additional culturally appropriate activities were investigated using recent field reports and personal communications with stakeholders in each location [80]. Squatting for toileting, sitting cross-legged to play games or eat, kneeling to pray or attend religious locations, and walking on an uneven terrain were identified as key parameters of function. Further activities were identified to be important to the rehabilitation of children with limb loss such as picking up objects from the floor and stepping over objects [81]. All resulting activity choices could also crucially be completed in any environment and do not require specialist equipment. Continuity across locations can be maintained. Therefore, the AMP format was used, and other culture and different environment activities incorporated.

#### Domain 4: Prosthetic design user requirements

User needs and prosthetic design requirements go hand in hand; however, it is challenging to derive the specific design requirements without gaining a discrete component level understanding of the user needs. Component level means that the user needs are directly related to the section of the prosthetic device (socket, knee etc.) that is either aiding or hampering the completion of their user need.

*Child*. Obtaining such requirements from children necessitates a short, fun, and approachable tool. Eleven tools were found and considered for analysis of this domain with the child [18,20–22,24–26,58,82–84], but none were found to be appropriate. Some were too long and complex for children and did not focus on specific components (OPUS; the Prosthesis Evaluation Questionnaire (PEQ); Satisfaction with Prosthesis Questionnaire, SAT-PRO; [24,25,82]. A focus group approach has also been used with adult lower-limb amputees [61,85,86], but these have not been applied to children. One group aimed to apply a multi-stakeholder focus group approach with this cohort, however no user requirement results have been published [87].

The majority of other prosthetic user questionnaires for children focussed on upper limb loss only [26,83]. Others focussed on very niche areas of provision such as active play or specific post prosthetic design concept evaluation [18–21,58]. Only one study was found which attempted to identify the user requirements of children with lower limb loss [88]. The research study was delivered in Cambodia and 3 children with limb loss and their families were interviewed using interactive activities like drawing. Although results of this study showed how children have their own unique requirements and aspirations, those are not generalisable as only a specific cohort was recruited (unilateral transtibial, congenital amputees). Additionally, as stigma against disability is a recognised problem in some countries researchers chose to focus only on cosmetic appearance and self-image, diverting answers towards a specific area [7,89,90]. Although this is a key prosthetic requirement, other desires children might have such as playing and running with their peers were overlooked and insight on their real priorities was not gained. The interactive nature of the interview played a key role in successfully delivering the study and should be a core aspect of developing research studies with children.

There is therefore no appropriate outcome measure, questionnaire, or interview guide suitable to meet the research objectives of this work. A novel measure is required to compliment the domains of function, QoL and pain whilst providing specific insight on children’s prosthetic design needs.

*Paediatric prosthetist*. The clinician who delivers and fits the prosthetic limb to the child (the prosthetist) is also a user of the device. They are involved with supply, fit and maintenance of the device. Their opinion and requirements are just as important, given they have expertise on technologies available, the needs of their patients and are familiar with the context, environment, and culture [17]. Previous studies interviewing prosthetists in high-income countries (HICs) and low resourced environments (LREs) have helped identify factors that prevent prosthetic usage or affect care in general [13–17,91]. However, there are no studies focussed on the paediatric prosthetist cohort.

Therefore, a novel interview guide for paediatric prosthetists must be developed which covers the necessary domains of care.

*Parent/Legal guardian*. Finally, as distinct from the adult with limb loss, for the child there is one more set of individuals in the user chain: the parent/guardian. The familial point of view cannot be omitted as their holistic understanding of the child’s care and rehabilitation is unparalleled [87]. The studies by Chhina et al. and Hussain et al. included families in the process as proxy (parent) reports are considered relevant for observable concepts and behaviours and can help to identify children’s priorities and potentially validate some of their answers [36,88,92].

**Fig 2:**
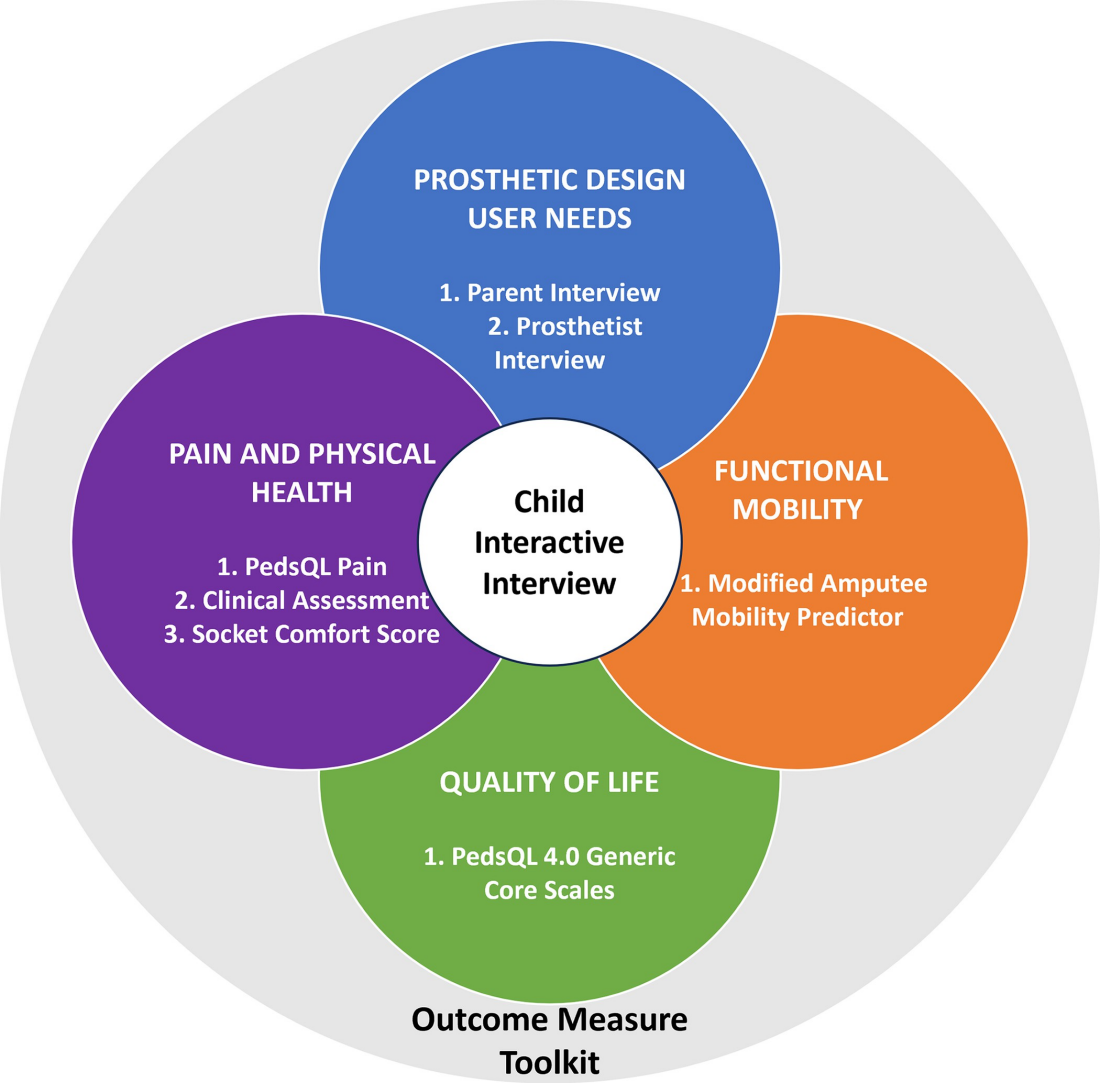
A toolkit for outcome measures for children with lower limb loss. The interactive interview guide for the child spans all four key domains and is placed central to the toolkit’s conceptual framework.

The user requirements discussion must therefore include these three stakeholders: child, parent/guardian, and paediatric prosthetist. In total 12 tools were considered for inclusion for which none met the objectives. To achieve this, 3 new measures must be developed to complement the other toolkit components. The result is a toolkit which provides comprehensive understanding covering all domains critical to the rehabilitation of a child with limb loss (Fig 2).

### Development of novel measures

#### Prosthetic user requirements content creation

**Fig 3:**
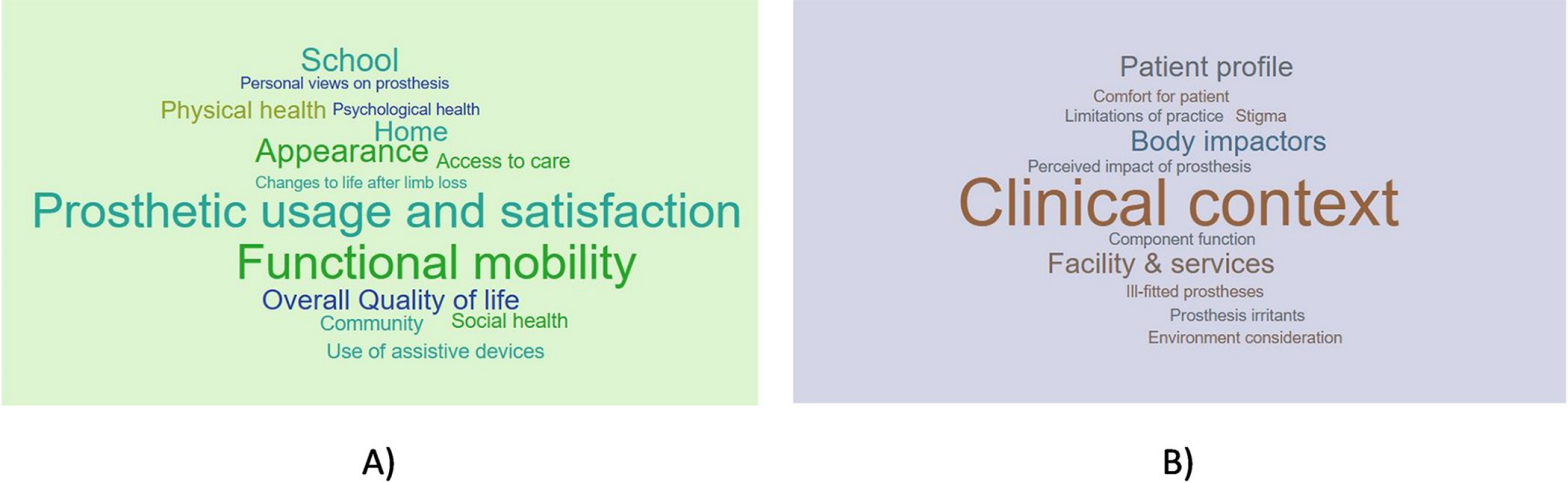
Themes for prosthetic user requirement interview guide development. A) Themes from literature on adult and paediatric user needs. B) Themes from literature on prosthetist interviews.

To create new measures for the user requirements domain, themes of interest were produced from an extensive literature review (Fig 3) [26,67]. The framework used by Hill et al. (2009) [83] was utilised as it showed a clear process to produce prosthetic outcome measures to directly inform and improve prosthetic design.

**Fig 4:**
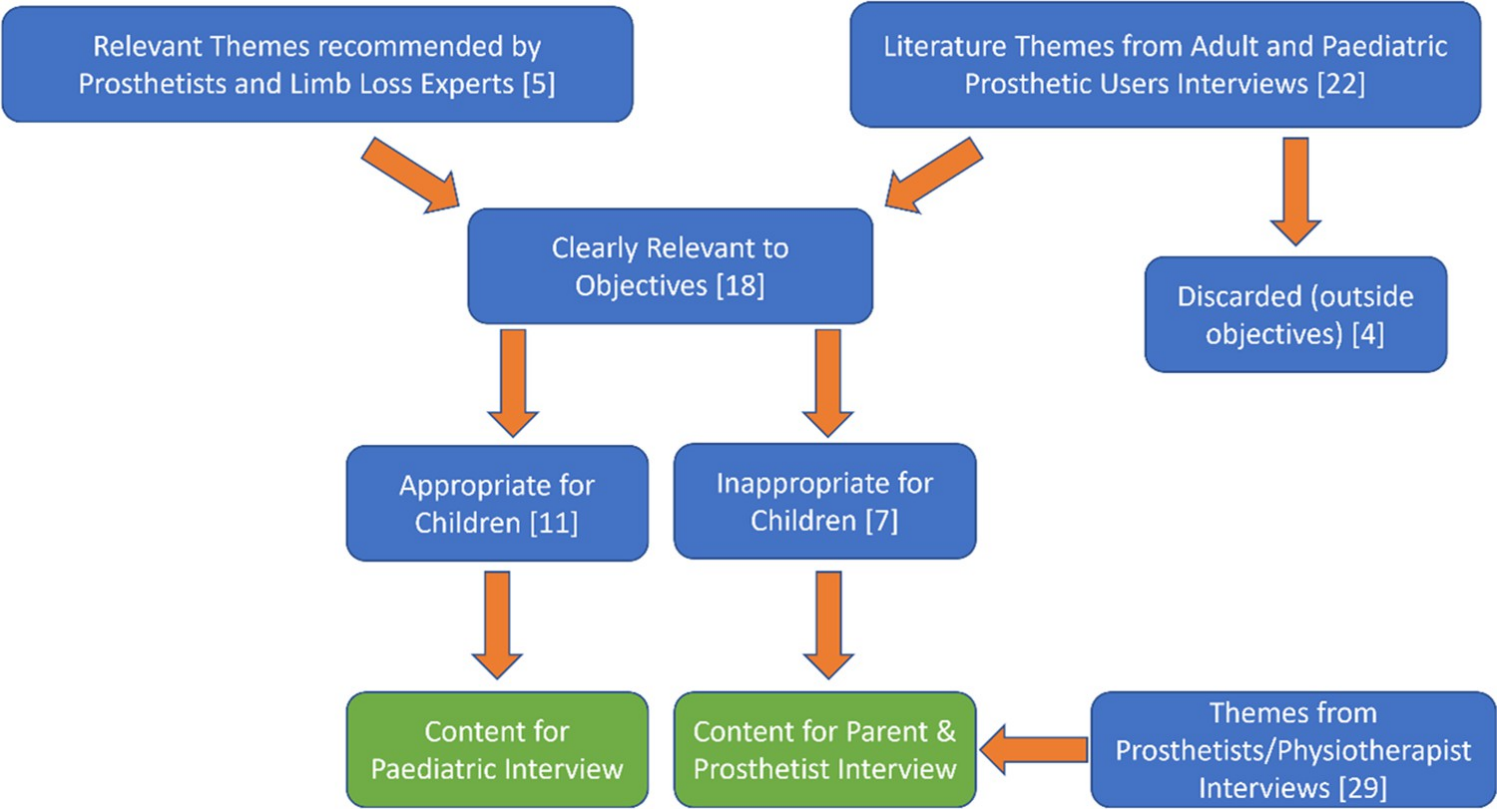
Thematic inclusion process. Brackets refer to numbers of themes identified through each method.

Once the themes had been identified, they were reduced and assessed by relevance and appropriateness for each user: child, parent/guardian, and prosthetist (Fig 4).

From the theme generation, content was first created for the child and then for the parents/prosthetists. This ensured all identified themes were included in the toolkit even if they were inappropriate for the child themselves to report on. Content was created for each theme at a component specific level so that discrete detailed design criteria could be elicited.

Age-appropriate interactive interview guides were developed for two age groups: 6–10 years and 11–18 years, following the recommendation from Bevans et al. (2010). Wording was chosen to be appropriately simple and clear, and each interview started with drawing activities to relax the child and gain insight through other means than pure questioning [22]. Drawings are used as a way to let the child show how they see themselves and their families, as well as their ‘dream’ prosthesis. This not only sheds light on their needs and vision, but also opens the possibility to delve into more detailed discussion of their ‘dream’ prosthesis.

**Fig 5:**
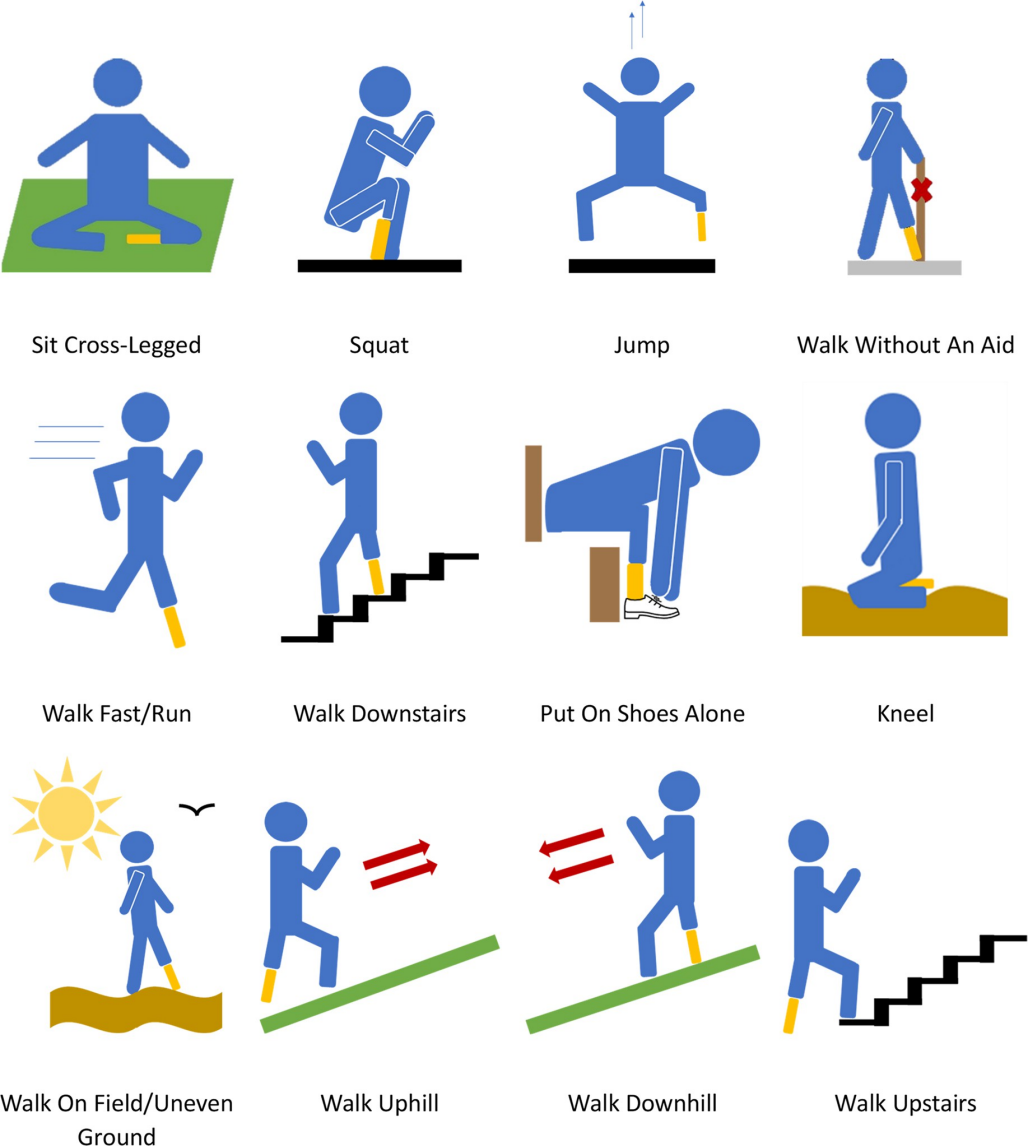
Card activity in interactive child interview guide. **“**For this next activity we would like you to split this set of cards into two groups, one group is the activities you can do, and the other group is the activities you cannot do. If you do not understand a picture, let me know and I will try and explain”.

Games and cards activities were created to provide both insight and a break from simple question and answer format. This method has been successfully used and recommended by researchers, an early year’s teacher, and a psychologist [88]. Examples of the card activities include choosing their favourite prosthesis in terms of cosmesis or ranking different concepts in order of importance (e.g., running fast, feeling less tired) (Fig 5). From age 6 years, children are expected to have the cognitive development to understand symbols and written words or numerals and thus this method would be appropriate for even the youngest participants [93]. Other recommendations by experts were to choose open-ended questions to give the child a chance to tell their own story and feel at ease.

**Fig 6:**
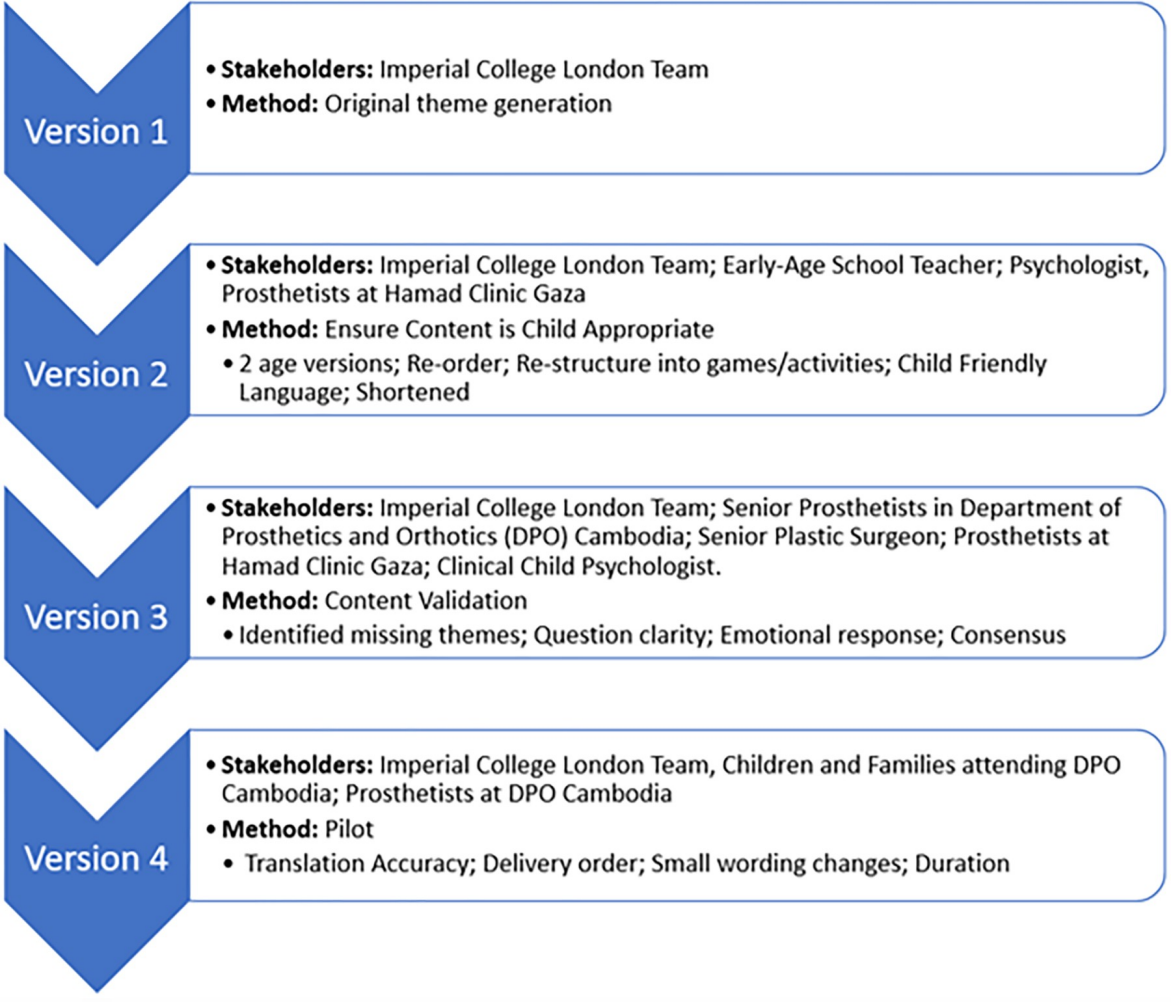
Flow-chart for child interview guide development–stakeholders and methods.

Each interactive interview guide was shortened to be appropriate to the age range with the chosen time limit of 15–20 minutes for below 10 years and 30–35 minutes for above 10 years, necessitating the removal of some themes (participant demographics, community living, rehabilitation process, clinical access). These were placed in the parent or prosthetist interview guide. Themes that were deemed too challenging or upsetting for the paediatric user themselves (e.g., changes to life after limb loss and details of amputation) as well as themes covered by the validated tools (e.g., social, and psychological health) were removed from the interview guide (Fig 6 Version 2). Themes from previous studies interviewing prosthetists or relevant to manufacturing and repairing prosthetics were only included in the prosthetist interview guide (Fig 3B) [13,16,17,94].

#### Delivery format

Interview guides for prosthetist and parent could follow the standard structured-interview format (duration: 60–90 minutes). However, specific considerations were necessary for the interaction with the child user. Instructions were inserted before and throughout the interactive interview guide to remind interviewers of key points such as prompts to look for physical and verbal signs of distress and discomfort (e.g., facial expression or resistance to answer) [12]. Reminders were left to be interactive and empathetic. Suggestions and recommendations on how to interact with children are present (e.g., ‘compliment their drawings,’ ‘mimic movement,’ ‘point at prosthetic component to explain’). Additionally, especially for conflict affected countries, they are made aware to check if the child is an orphan and if so to skip the ‘draw you and your family’ activity. These points are also highlighted in an extensive 2-hour training session before data collection. These aspects are critical to both the successful distillation of information as well as the enjoyment for the child in partaking in research.

The choice of games and card-based activities was key. They allow the child to take their time, play with the different options and visualise the options clearly. The end result is a discrete choice or ranking which provides quantitative results and opens the door to further discussion of ‘why did you pick that card?’ Additionally, they provide easy flexibility for different cultural/geographical environments. New card options can be added in/removed based on the context providing an effective repeatable method with a high level of flexibility.

#### Content and delivery format validation–expert panel review

Following content creation, a panel of experts in paediatric limb loss and child mental health independently reviewed the questions and provided feedback on missed areas. Multi-stakeholder content validity is typically achieved in this way through an iterative process [25,26,39,67,86]. The panel consisted of three senior prosthetists at the Department of prosthetics and orthotics (DPO), a prosthetist at the Hamad clinic Gaza, four senior researchers within the field of paediatric limb loss and traumatic injury and a child mental health professional. Feedback consisted of missing questions, wording, and style of question (Fig 6 Version 3). The resulting interview guide was reviewed by the child mental health professional who has worked with children from age 5–18 years displaying a variety of mental health challenges and experiences. It was required that they agreed to the inclusion and wording of every question within the document. They provided essential improvements and confidence that this vulnerable population would not be emotionally triggered by any of the interview processes. Timing of final versions was assessed by simulating the interview setting.

#### Pilot testing

The final toolset contained:

1. The novel interview guides for the child, their parent/guardian and treating prosthetist,
2. A modified version of the Amputee Mobility Predictor,
3. The validated PedsQL Pain and Quality of Life and Socket Comfort Score measures,
4. Clinical evaluation form.

The authors combined validated and novel measures in a toolset to meet the study objectives as multiple previous studies had successfully achieved this [95,96].

The toolkit went through 5 pilot tests across the 3 centres in Cambodia to evaluate both age versions of the interview guides. This provided key validation and allowed the children to become stakeholders in the study design process.

The full toolset was translated by the DPO into Khmer. The experience of the individuals at DPO who clinically work in this field ensured key medical terminology was translated correctly. The full toolset was analysed by a senior lecturer at DPO, who checked 3 aspects.

1. Suitability of delivery format to the environment,
2. Suitable duration,
3. Relevance and comprehensibility of questions for each age group.

The pilots were conducted across three prosthetic centres of the DPO to ensure location was not a confounding factor (Phnom Penh, Kampong Chhnang, Kampong Som). The pilots were conducted with girls and boys from a range of ages (Table 3).

**Table 3:** Demographics of pilot participants and interview timings.

Number	Age Range (yrs)	Sex	Level of Amp	Timing (mins)
**Iteration n˚ 1**				
**Pilot_01**	10–18	F	Transtibial	Child: 45
**Pilot_02**	<10	F	Bilateral Transfemoral	Child: 35
**Iteration n˚ 2**				
**Pilot_03**	10–18	M	Transtibial	Child: 60
**Pilot_04**	10–18	M	Transtibial	Child: 60
**Pilot_05**	<10	F	Transtibial	Child: 60

Each pilot provided critical feedback to the project for both content and protocol, however the toolset was very positively received, and only small changes were required. Any questions which were challenging to understand were rechecked in terms of translation and then any required rewording applied. If other unexpected topics were raised during the pilot, consideration of additional questions was conducted. For example, a younger child showed clearer understanding of their different prosthetic componentry and was able to provide distinct feedback on the knee joint, so a more specific question was added to the below 10 years version.

The parent and prosthetist tools went through the same process with 5 parents and 2 prosthetists. They were additionally piloted as a written questionnaire instead of an interview to check the impact on data quality. In future, they should be conducted as an interview to maintain depth of data. In cases where duration of delivery is further limited and illiteracy is not a potential issue, these two guides may be delivered through a written format. The child interview should always be delivered in person to maintain the interactive nature. It is now believed the toolset produced through this process is effective and sufficiently analyses the domains of interest (Fig 6 Version 4).

### Patient and public involvement

Key stakeholders including patients and the public were involved throughout this work. As discussed, experts in research and care for children with limb loss were involved in the development of novel interview guides and the validation of content. Additionally, toolkit piloting with children, and extensive conversations with clinical teams in each geographical location informed the study design plan and final delivery protocol detailed below.

## Study delivery

### Ethics and dissemination

This Study has ethical approvals from:

- National Ethics Committee for Health Research (N˚ 024NECHR) of Cambodia on 18^th^ of January 2023.
- Helsinki Committee of Gaza (PHRC/HC/973/21) on 11/10/2021,
- American University of Beirut Research Ethics Committee (SBS-2022-0368) on 5^th^ of October 2023,
- Wales Research Ethics Committee (REC Reference: 21/WA/0027) on 12^th^ of May 2023, and
- Imperial College London Research Ethics Committee (N˚ 6609720) on 04^th^ of August 2023.

Results will be disseminated through manuscripts in academic journals and presentations at academic conferences.

### A priori sample size estimation

A large challenge when determining an a priori sample size for this population is the scarcity of existing work in this area and the niche cohort [97]. Additionally, the methodology employed for this study is mixed with both qualitative and quantitative aspects. As such, each merits its own consideration for sample size. The sample sizes below represent the numbers required in each geographical location.

### Quantitative measures

Due to wide use of PedsQL 4.0 Generic Core Scales with different cohorts of children, sample size calculations were based on the Total Composite Score (TCS) of this questionnaire [45,48,98]. TCS from healthy children ($\bar{x}$ = 82.87 ± 13.16) and children with cerebral palsy (a significantly debilitating musculoskeletal disorder) ($\bar{x}$ = 66.5 ± 16.73) were used to estimate the sample size necessary to provide >80% power at an alpha of 0.05 (two sided) [46,48]. A cohort with another musculoskeletal condition was chosen due to a lack of available results for a paediatric limb loss cohort. A sample size of 11 was predicted.

### Qualitative interviews

Qualitative research has shown from an analysis of 60 interviews that 73% of thematic codes were identified in the first 6 interviews and 92% within the first 12 [99]. Based on this a minimum of 6 children from each lower-limb amputation level in each location was selected to provide an in depth and representative analysis.

### Planned statistical analysis plan

Demographics, QoL, pain and functional mobility measures of the cohort will be reported with descriptive statistics to identify any potential trends across results (e.g., QoL and gender, functional mobility for different amputation levels). Correlations will be explored with linear regression models using independent-sample t-tests and Pearson correlation. These tests will also be used to assess differences in QoL of children living in the 3 contrasting study locations to assess the impact of prosthetic provision has on this key measure (e.g., high-tech componentry versus lower quality ones).

Qualitative data will be analysed following the reflexive thematic analysis principles laid out by Braun and Clarke [100]. The interviews will be translated and transcribed in English by the local research team, where relevant. They will be coded into themes utilising a deductive approach led by the objectives of the study. Taking inspiration from Maslow’s hierarchy of needs, these themes will then be used to create a hierarchy of user needs and technical design features based on their prevalence within the data [101]. A hierarchy of needs will streamline the prosthetic design innovation process ensuring researchers include at least the most important features. This has been previously applied in the area of Type 1 diabetes which shows how this inherent concept can provide clear insight into the most important aspects of a condition [102]. A separate hierarchy of needs will be produced for each user for each location to show contrasting and consistent results. Data collection will continue until the targeted number of subjects is recruited. Data collection in each location is expected to last a maximum of one year to ensure environmental factors and clinical practice do not change significantly between subjects.

## Learnings and ethical considerations for research with children

Children with disabilities are a vulnerable cohort. It is key to make sure ethical processes are followed to both provide these users a voice in a safe environment but also respect their dignity and autonomy. Although challenging to design and develop a study with this cohort, the field should not shy away from giving this key user base a platform to share their views through ethical study design. The following section provides the authors’ experience during the development and pilot of the toolkit as advice for future research in the field of paediatric assistive technologies. This work was structured following the steps recommended by Johnson et al. [12].

### Step 1: Consideration of Capacity and Capability

It is essential to remember limitations in children’s attention span. This time constraint means there is a limited amount of the data that can be collected to ensure data quality is maintained and the participant is not overwhelmed. It is important to gauge what information is key to ask children directly and what can be simply reported by legal guardians. The researcher may not be able to achieve everything they initially set out to include, and so compromise is essential. Enjoyment of the research process is paramount, their rehabilitation as children with limb loss has significant challenges they must overcome daily, and the research should not add to these challenges.

### Step 2: Developing Ethical Protocols and Processes

Part of ethical design includes ensuring an appropriate consent process is followed [103] and this study requires both adult consent and child assent. The principle of Gillick Competency is applied to enrol children within a research study. First the study is clearly explained in lay language to the legal guardian/parent of the child before written consent is gained. Only after this, the child is approached with a simple yet comprehensive explanation of the study using an age-appropriate participant information sheet. Time to ask any questions is given and the right to stop at any time is repeated multiple times. Assent is gained while asking the child in an open and unpressurised manner if they would like to take part.

### Steps 3,4,5: Developing trust and relationships; selecting appropriate methods and identifying appropriate forms of communication

Children are not small adults: approaching and interacting with them is very different from interviewing an adult. Activities should be fun and interactive, and attention should be given to avoid triggering traumatic experiences. Interviewers must be trained to gain their trust before starting the research study as well as appropriately communicate with them throughout the interview. Extensive time was dedicated to developing appropriate content and delivery format as outlined in the section ‘Development of Novel Measures.’ Content can be adapted to reflect the needs of the cohort of interest and the research question, but the methodology and best practice can still apply. Further considerations such as sign language translation for hearing loss or more extensive use of the card games depending on cognitive capacity can be included.

### Step 6: Consideration of Context

Different countries have different lifestyles, culture and habits and the study content should adapt to maintain appropriateness as well as respect for different cultures and resources available. Current study format allows for interchangeability and flexibility of content (e.g., changing activities on cards based on relevance) whilst maintaining the overall aim and structure. Questioning must consider potential sensitivity or stigma around disability. Children may feel uncomfortable discussing certain aspects of body image or community integration. Methods to elicit information without causing emotional upset included asking about the child’s favourite clothes rather than questioning directly on body image. This opens up the correct dialogue without pressure. Local research teams have been involved since the beginning to ensure the content and questions were relevant to the culture and context, and no key area was omitted. These local teams will deliver the study and conduct data collection. The clinical and research teams on the ground have expertise and knowledge that far surpasses anyone else and the success of any work in a clinical environment relies on this understanding [30].

To conclude, participant data protection is important and even more so when working with a vulnerable cohort. Data will be pseudo-anonymised to ensure anonymity. Data will be shared across institutions through a secure, password-controlled folder.

## Supporting information

S1 File Functional movement assessment. (PDF)

S2 File Parent interview guide. (PDF)

S3 File Prosthetist interview guide. (PDF)

S4 File Questionnaire children above 10. (PDF)

S5 File Questionnaire children below 10. (PDF)
