# Supplementary material for: Listening to children with lower limb loss: Rationale, design, and protocol for delivery of a novel globally applicable research toolkit—Prosthetic user needs, quality of life, pain, and physical function
Source: PLoS One. 2024 Oct 31;19(10):e0310848. doi: 10.1371/journal.pone.0310848 (PMC11527159; doi:10.1371/journal.pone.0310848)
Supplement: S1 File — (PDF) [file pone.0310848.s001.pdf]

Patient ID: ……………………………………

Functionalities Test

The aim of this test is to understand the function of the child with limb loss in completing standard tasks included in typical rehabilitation at the clinic. All or some variation in the tests below can be included depending on resources and the guidance of the treating clinician. If visual confirmation of any of these activities occurs at any point during the interview process, feel free to grade them without the need to repeat such as walking freely.

Date of Test: Day ……… Month ……… Year …………

Test 0: Standing Balance

Define the completion of activity	Additional Notes
☐ **(0) Cannot stand upright**<br>☐ **(1) Can stand upright with aid/support**<br>☐ **(2) Can stand independently for a few seconds**<br>☐ **(3) Can stand independently for extended period**	

Test 1: Walking between parallel bars

Define the completion of activity	Additional Notes
☐ **(0) Cannot walk**<br>☐ **(1) Walking holding both rails**<br>☐ **(2) Walking holding one rail**<br>☐ **(3) Walking freely with no rail support**	

Test 2: Walking Freely

Define the completion of activity	Additional Notes
☐ **(0) Cannot walk**<br>☐ **(1) Walking with crutches**<br>☐ **(2) Walking with a walking aid/support of parent**<br>☐ **(3) Walking freely with no support**<br>☐ **(4) Walking freely with no support and variable cadence**	

Patient ID: ……………………………………

Test 3: Sitting down on Bench/Chair

Define the completion of activity	Additional Notes
☐ (0) Cannot sit down independently<br>☐ (1) Sit down using two hands<br>☐ (2) Sit down using one hand<br>☐ (3) Sit down using no support	

Test 4: Standing up from a Bench/Chair

Define the completion of activity	Additional Notes
☐ (0) Cannot sit down independently<br>☐ (1) Sit down using two hands<br>☐ (2) Sit down using one hand<br>☐ (3) Sit down using no support	

Test 5: Walking up ramp

Define the completion of activity	Additional Notes
☐ (0) Cannot walk up ramp<br>☐ (1) Walk up ramp holding both rails<br>☐ (2) Walk up ramp holding one rail<br>☐ (3) Walk up ramp freely with no rail support	

Test 6: Walking down ramp

Define the completion of activity	Additional Notes
☐ (0) Cannot walk down ramp<br>☐ (1) Walk down ramp holding both rails<br>☐ (2) Walk down ramp holding one rail<br>☐ (3) Walk down ramp freely with no rail support	

Patient ID: ....

Test 7: Walking upstairs

Define the completion of activity	Additional Notes
☐ (0) Cannot walk upstairs<br>☐ (1) Walk upstairs holding both rails<br>☐ (2) Walk upstairs holding one rail<br>☐ (3) Walk upstairs freely with no rail support	

Test 8: Walking downstairs

Define the completion of activity	Additional Notes
☐ (0) Cannot walk downstairs<br>☐ (1) Walk downstairs holding both rails<br>☐ (2) Walk downstairs holding one rail<br>☐ (3) Walk downstairs freely with no rail support	

Test 9: Walking on/over obstacle

Define the completion of activity	Additional Notes
☐ (0) Cannot attempt<br>☐ (1) Step on/over 1 step with support<br>☐ (2) Step on/over 1 step without support<br>☐ (3) Step on/over 2 steps with support<br>☐ (4) Step on/over 2 steps without support<br>☐ (5) Step on/over more than 2 steps with support<br>☐ (6) Step/over on more than 2 steps without support	

Test 10: Walking on Uneven Ground

Define the completion of activity	Additional Notes
☐ (0) Cannot walk<br>☐ (1) Walking with crutches<br>☐ (2) Walking with a walking aid/support of parent<br>☐ (3) Walking freely with no support	

Patient ID: …………………………………

Test 11: Sitting down on the ground

Define the completion of activity	Additional Notes
☐ (0) Sitting down with parent helping<br>☐ (1) Sitting down on ground with support such as chair<br>☐ (2) Sitting down on their own	

Test 12: Kneeling

Define the completion of activity	Additional Notes
☐ (0) Cannot kneel<br>☐ (1) Kneeling with quadriceps at 90 degrees to floor<br>☐ (2) Kneeling with quadriceps around 45 degrees to floor<br>☐ (3) Kneeling with weight on heels of feet (full range of motion)	

Test 13: Sitting Cross-Legged

Define the completion of activity	Additional Notes
☐ (0) Unable to bend knees<br>☐ (1) Bending knee at all during sitting<br>☐ (2) Sitting cross legged	

Test 14: Pick up object from floor

Define the completion of activity	Additional Notes
☐ (0) Cannot pick up object<br>☐ (1) Can pick up object with support (chair/parent)<br>☐ (2) Can pick up object independently	

Test 15: Squatting

Define the completion of activity	Additional Notes
☐ (0) Cannot squat<br>☐ (1) Can only squat with the intact leg<br>☐ (2) Can partially squat with both legs (both knees partially flexed less than 20˚)<br>☐ (3) Can properly squat with both legs (knees fully flexed)	
