# Supplementary material for: Listening to children with lower limb loss: Rationale, design, and protocol for delivery of a novel globally applicable research toolkit—Prosthetic user needs, quality of life, pain, and physical function
Source: PLoS One. 2024 Oct 31;19(10):e0310848. doi: 10.1371/journal.pone.0310848 (PMC11527159; doi:10.1371/journal.pone.0310848)
Supplement: S2 File — (PDF) [file pone.0310848.s002.pdf]

Participant ID: ……………………………

Parent/Guardian Questionnaire for a child with an amputation

This is a questionnaire which asks about your knowledge and feelings about your child's amputation as well as the feelings and function you believe your child currently has. You are free to answer as many of these questions as you can or feel comfortable doing. Please take your time and if a question does not make sense please check with the interviewer.

Please fill in where requested and tick the relevant boxes.

Example → 12-year-old boy from a rural village who lost is leg 2 years ago due to gangrene is enrolled in the study. Section 1 would be filled like this:

Example Section 1: Participant's Demographics

Child demographics

Child's Date of Birth:	Day: 05 Month: 05 Year: 2011
Gender at Birth:	☐ Female
	☒ Male
Residence:	☒ Rural
	☐ Urban
Date of Amputation:	Day: 06 Month: 07 Year: 2020
Cause of Amputation:	
	☐ Congenital<br>☐ Cancer<br>☒ Infection<br>☐ Trauma<br>☐ Other (please write) ………………………
If Trauma, state type:	☐ Conflict<br>☐ Non-conflict

If Non-Conflict, please specify cause: ……………………………………

If Conflict, state type:	☐ Blast<br>☐ Gunshot<br>☐ Other (please write): …………..

Participant ID: ………………………….

Section 1: Participant's Demographics

Child demographics

Child's Date of Birth: Day: ………. Month: ………. Year: …………

Gender at Birth: ☐ Female

☐ Male

Residence: ☐ Rural

☐ Urban

Date of Amputation: Day: ………. Month: ………. Year: …………

Cause of Amputation:

☐ Congenital
☐ Cancer
☐ Infection
☐ Trauma
☐ Other (please write) ……………………….

If trauma, state if conflict related: ☐ Conflict
☐ Non-conflict

If Conflict, state type: ☐ Blast
☐ Gunshot
☐ Other (please write): …………..

If Non-Conflict, please specify cause of trauma: ……………………………………

Parent/Guardian demographic

Date of Birth: Day: ………. Month: ………. Year: …………

Gender at Birth: ☐ Female

☐ Male

Caregiver's marital status: ☐ Single

☐ Married

☐ Separated

☐ Divorced

☐ Widowed

Relationship to the child: ☐ Mother

☐ Father

☐ Step/foster-mother

☐ Spouse

☐ Sibling (brother/sister)

☐ Other (please write) ....

What is your occupation? ....

Section 2: Amputation experience and daily life challenges

1. Does your child currently use a prosthetic limb? ☐ Yes ☐ No
2. At what age did your child receive their first prosthetic limb? Please write ....
3. Has your child received or used any other assistive devices throughout their limb loss?

   ☐ Wheelchair: ...... Age: .... To: ....

   ☐ Crutches: .... Age: .... To: ....

   ☐ Cane: .... Age: .... To: ....

   ☐ Other (please write) .... Age: .... To: ....
4. Is your child able to walk independently on their prosthesis without the use of an assistive device? ☐ Yes ☐ No
5. Do you ever carry your child? ☐ Yes ☐ No
   a. ***If yes,*** when?

....

6. How does your child feel about their appearance with their limb loss?

....

....

7. How do you feel about your child's appearance with their limb loss?

....

....

8. Does your child ever get angry or sad with their prosthetic? ☐ Yes ☐ No
   a. ***If yes,*** when?

....

....

9. ***If not congenital limb loss.*** Has your child changed the way they like to dress since their limb loss?

....

....

Participant ID: ……………………………

Section 3: Clinical

1. When did your child first access the prosthetic rehabilitation clinic?

   Day: ……. Month: ….. Year: ……..

2. Was there any delay in your child receiving a prosthesis? ☐ Yes ☐ No
   a. ***If yes,*** how long and why?

………………………………………………………………………………………………………

………………………………………………………………………………………………………

3. Which mode of transport do you usually use? ……………………………………………
4. Financial cost to travel to the clinic in general/last time you were here:

Item	Cost
Transport	
Accommodation	
Food and other miscellaneous	

   a. How many people are included in the cost detailed above (e.g. you and your child; you, your spouse, and your child)? …………………………………………

5. Time to travel to the clinic:

Item	Specify appropriate unit of time (minutes, hours, days)
Time to travel to the clinic	
Night spent at the accommodation	

   a. How many people are included in the cost detailed above (e.g. you and the child, you, your spouse, and your child)? …………………………………………
   b. If you are at the clinic for a prosthetic replacement, do you usually stay in an hotel/accommodation or do you travel back and forth from home?

………………………………………………………………………………………………………

   c. Why do you choose to do so?

………………………………………………………………………………………………………

………………………………………………………………………………………………………

6. Who usually brings your child to the clinic? ………………………………………………..

Participant ID: ……………………………

7. Do you/they have to take time off work/school to come to the clinic?

………

………

8. How long does a clinic visit usually last? …………………………………………………
9. How long do you have to wait usually between a casting visit and a fitting visit?

………

10. How many times a year does your child go to the clinic? ………………………………
11. Why does your child usually need to return to the clinic? What is the cause?

………

………

12. How frequently is the prosthetic leg replaced?

………

13. Are you happy with the service you receive at the clinic?

………

………

14. Do you pay for some or all the prosthetic devices your child receives?
    a. ***If fully covered by insurance/government***, how much would you be able to pay for a prosthetic leg (e.g., $50)?

………

15. Does your child wish they could visit the clinic less often?

………

………

16. Has your child required any revision surgeries post-amputation?

………

………

Participant ID: ……………………………

Section 4: Mobility and Prosthesis Use

1. How many days in the last week has your child used their prosthetic limb or assistive device?

   ☐ Prosthetic ……………………

   ☐ Assistive device: ……………..

2. How many hours in a day does your child normally use their prosthetic or assistive device?

   ☐ Prosthetic ……………………

   ☐ Assistive device: ……………..

3. What causes you or your child to remove their prosthetic limb during the day?

………………………………………………………………………………………………………

………………………………………………………………………………………………………

4. Does your child wear their prosthetic limb in public, i.e., to school, shops, church, temple, mosque etc? ☐ Yes ☐ No

   a. Please explain when?

………………………………………………………………………………………………………

………………………………………………………………………………………………………

5. Do they wear their prosthetic at home? ☐ Yes ☐ No

   a. Please explain why?

………………………………………………………………………………………………………

………………………………………………………………………………………………………

   b. ***If yes***, do they wear it uncovered? ……………………………………………

6. How does everyone typically sit down at home or at school or at a religious place?

………………………………………………………………………………………………………

This next activity asks you to state whether your child is able to complete each of the activities and give additional explanation on each activity such as how comfortable they are when completing the activity, how often they complete the activity or any other information you think will be useful to note down. *(Add in other activities that are relevant to environment of use).*

Participant ID: ……………………………

Activity	Yes/No	Please explain
Walk independently using their prosthesis without a cane/crutch	☐ Yes<br>☐ No	
Walk upstairs	☐ Yes<br>☐ No	
Walk downstairs	☐ Yes<br>☐ No	
Walk uphill	☐ Yes<br>☐ No	
Walk downhill	☐ Yes<br>☐ No	
Run	☐ Yes<br>☐ No	
Sit on the ground	☐ Yes<br>☐ No	
Squat	☐ Yes<br>☐ No	
Kneel	☐ Yes<br>☐ No	
Walk on uneven terrain	☐ Yes<br>☐ No	
Walk on wet ground/ walk when it has been raining	☐ Yes<br>☐ No	
Bathe/Shower on their own	☐ Yes<br>☐ No	
Put on their shoes on their own	☐ Yes<br>☐ No	
Dress on their own	☐ Yes<br>☐ No	
Put on their prosthetic limb on their own	☐ Yes<br>☐ No	

Participant ID: ……………………………

Wear appropriate traditional or religious clothing	☐ Yes<br>☐ No	
Walk as fast as they want	☐ Yes<br>☐ No	
Sit with the legs crossed	☐ Yes<br>☐ No	
Toileting	☐ Yes<br>☐ No	

7. What activities does your child talk about wanting or wishing they could do the most?

………………………………………………………………………………………………………

………………………………………………………………………………………………………

8. How long can your child walk before needing a rest?

………………………………………………………………………………………………………

Section 5: Prosthetic Componentry

1. How long does it take for your child to get used to a new prosthetic limb iteration?

………………………………………………………………………………………………………

………………………………………………………………………………………………………

2. If the prosthetic leg needed fixing, would you bring it back to the clinic or try fix it yourself?

………………………………………………………………………………………………………

………………………………………………………………………………………………………

3. Was your child taught how to use their prosthetic device or given any rehabilitation?

☐ Yes ☐ No

   a. What did you think of this process?

………………………………………………………………………………………………………

………………………………………………………………………………………………………

Participant ID: ……………………………

4. Do you clean the prosthetic? ☐ Yes ☐ No
   a. ***If yes,*** where, and how?

………………………………………………………………………………………………………
………………………………………………………………………………………………………

5. Is your child generally happy with their current prosthetic? ☐ Yes ☐ No
   a. Please expand on this

………………………………………………………………………………………………………
………………………………………………………………………………………………………

6. What does your child tell you they wish they could change about their leg?

………………………………………………………………………………………………………
………………………………………………………………………………………………………

7. Does your child fall over often?

………………………………………………………………………………………………………
………………………………………………………………………………………………………

8. Does your child ever complain about any noise their prosthetic makes?

………………………………………………………………………………………………………
………………………………………………………………………………………………………

This next section talks about specific components that make up your child's prosthetic leg, if you do not know which component is which, please let me know and I can show you on an example leg. Some components will not be relevant to your child due to their level of limb loss, please skip these sections where appropriate (e.g., Skip the knee joint if your child has a below knee limb loss).

The first component we are going to talk about is the socket that goes over your child's residual limb.

9. What issues, if any, does the prosthetic socket cause your child?

………………………………………………………………………………………………………
………………………………………………………………………………………………………

Participant ID: ……………………………

10. Does the socket cause you and your child to go to the clinic more often?

……
……

11. Does the socket cause your child to take off their prosthetic limb?

……
……

12. Does the socket cause any skin problems on their residual limb?

……
……

The second component we are going to talk about is the prosthetic knee that joins the socket to the shank section of your child's limb. This is only applicable if your child has limb loss above or through the knee joint.

13. Does your child have a prosthetic knee joint? ☐ Yes ☐ No
    a. ***If no,***
        i. Do you know why not?

……
……

        ii. Do you think a prosthetic knee would be helpful for your child?

……
……

14. Is your child able to bend their knee? ☐ Yes ☐ No
    a. ***If yes,***
        i. Is it helpful for the knee to bend?

……
……

        ii. Does your child feel stable on their knee?

……
……

        iii. Are you happy with the appearance of the knee?

……
……

iv. Is there anything you would change about their movement or function around their knee?

……………………………………………………………………………………………………

……………………………………………………………………………………………………

v. Has the knee ever got caught in their clothes?

……………………………………………………………………………………………………

……………………………………………………………………………………………………

vi. Has the knee ever broken or required repair?

……………………………………………………………………………………………………

……………………………………………………………………………………………………

b. ***If no,***

i. Would it be helpful for your child to bend their knee?

……………………………………………………………………………………………………

……………………………………………………………………………………………………

ii. Does your child feel stable on their leg?

……………………………………………………………………………………………………

……………………………………………………………………………………………………

The third component we are going to talk about is the foot/blade.

15. Does your child use a prosthetic foot? ☐ Yes ☐ No

a. If yes, when do they choose to wear their foot?

……………………………………………………………………………………

……………………………………………………………………………………

b. What problems, if any, does the prosthetic foot cause?

……………………………………………………………………………………

……………………………………………………………………………………

c. Does your child like the appearance of the foot?

……………………………………………………………………………………

……………………………………………………………………………………

d. Is there anything you would change about the foot?

……………………………………………………………………………………

……………………………………………………………………………………

e. Does the foot usually break, if so, how?

……………………………………………………………………………………

……………………………………………………………………………………

16. ***If in a high resourced environment:*** Does your child use a running blade?

☐ Yes ☐ No

a. If yes, when do they choose to wear their blade?

……………………………………………………………………………………

……………………………………………………………………………………

b. What problems, if any, does the blade cause?

……………………………………………………………………………………

……………………………………………………………………………………

c. Does your child like the appearance of the blade?

……………………………………………………………………………………

……………………………………………………………………………………

d. Is there anything you would change about the blade?

……………………………………………………………………………………

……………………………………………………………………………………

e. Does the blade usually break, if so, how?

……………………………………………………………………………………

……………………………………………………………………………………

Section 6: Discomfort

1. Does your child struggle with sweating in their socket? How do you try and solve this?

……………………………………………………………………………………………

……………………………………………………………………………………………

2. Does pain ever cause your child to remove their prosthetic/assistive device?

……………………………………………………………………………………………

……………………………………………………………………………………………

3. Where in their body is your child most tired by the end of the day?

……………………………………………………………………………………………

……………………………………………………………………………………………

Participant ID: ……………………………

Section 7: Community

1. Does your child go to school? ☐ Yes ☐ No
   a. ***If yes,***
      i. Does your child enjoy school? ☐ Yes ☐ No
         1. Please expand:

……………………………………………………………………..…………………………………

………………………………..……………………………………………………………………

         2. How many days of school in a month does your child skip because of prosthesis' issues or visits to the prosthetic clinic?

            …………………………………………………………………………...

         3. What is the most common reason why your child skips school?

……

……

   b. ***If no,***
      i. Why not?

……

……

      ii. Did your child go to school before they lost their limb?

……

      iii. Is your child educated at home?

……
