# Supplementary material for: Listening to children with lower limb loss: Rationale, design, and protocol for delivery of a novel globally applicable research toolkit—Prosthetic user needs, quality of life, pain, and physical function
Source: PLoS One. 2024 Oct 31;19(10):e0310848. doi: 10.1371/journal.pone.0310848 (PMC11527159; doi:10.1371/journal.pone.0310848)
Supplement: S3 File — (PDF) [file pone.0310848.s003.pdf]

Participant ID:

Questions for a Prosthetist at P&O centre:

Please ask if they are okay being audio-recorded. If possible, please record them.

This is a questionnaire used to assess the overall provision from a prosthetic centre who provide for children with limb loss. It should be used as a guide for conversation.

Name of Centre: …….

Location of Centre: ………

Date of Interview (DD/MM/YYYY): ………………………………………………………………………………………

Section 1: Paediatric Prosthetic Provision, demographics, and key challenges

1. For how many years have you been working with paediatric amputees?
    a. Please specify here: ……………………………….
2. Roughly how many paediatric lower-limb amputees do you see in one year?
    a. Please specify here: ……………………………….
3. What levels of lower limb amputation do you provide for paediatric lower-limb amputees? Tick all that apply.
   ☐ Partial Foot Amputation
   ☐ Ankle Disarticulation
   ☐ Transtibial/Below Knee
   ☐ Knee Disarticulation
   ☐ Transfemoral/Above Knee
   ☐ Hip Disarticulation
4. Do you treat children of all ages? ☐ Yes ☐ No
5. What is the youngest age you will provide a prosthetic limb? Please specify: ………………………
6. Do your lower-limb paediatric amputee patients live in urban areas or in rural areas? Please tick the correct box.

   ☐ All in rural

   ☐ Most rural

   ☐ About equal

   ☐ Most urban

   ☐ All urban

7. What is the most common cause of childhood amputation?
   ………
    a. Circle the correct amount of children that attend due to the following causes of amputation.

   Congenital:
   ☐ All ☐ Most ☐ Some ☐ Very Few ☐ None
   Cancer:
   ☐ All ☐ Most ☐ Some ☐ Very Few ☐ None
   Infection/disease:
   ☐ All ☐ Most ☐ Some ☐ Very Few ☐ None

Trauma:

☐ All ☐ Most ☐ Some ☐ Very Few ☐ None

Other (please specify): ………………………………………………

☐ All ☐ Most ☐ Some ☐ Very Few ☐ None

8. With traumatic amputation, what is the most common cause? …………………………
    a. Circle the correct statement for each of the following event that can cause a traumatic amputation.

Road Traffic Accident:

☐ All ☐ Most ☐ Some ☐ Very Few ☐ None

Other Accident (E.g., Falling from tree):

☐ All ☐ Most ☐ Some ☐ Very Few ☐ None

Blast Injury:

☐ All ☐ Most ☐ Some ☐ Very Few ☐ None

Gun Shot Wound:

☐ All ☐ Most ☐ Some ☐ Very Few ☐ None

Other (please specify): ………………………………………

☐ All ☐ Most ☐ Some ☐ Very Few ☐ None

9. How often do your lower-limb paediatric amputee patients visit the centre?

    ☐ Every month

    ☐ Every three months

    ☐ Every six months

    ☐ Once a year

    ☐ Other (please specify): ……………………………………………………………………….

10. What is the main reason your lower-limb paediatric amputee patients return to the centre if the appointment is not scheduled?

…….

11. What challenges do you face in your paediatric prosthetic provision?

……

    a. Out of those challenges, which would you say is the largest or most challenging? ……….
    b. Please rank the challenges you have given in order of importance. If the below are not mentioned, ask if they are challenges, or if just not important challenges.

        ☐ Limited resources
        ☐ Insurance
        ☐ Child growth
        ☐ Limited componentry for paediatric lower-limb amputees
        ☐ Supply chain delays

☐ Other (please specify): ……………………………………………………………………….

12. What is your top priority for future development in paediatric prosthetic provision? What would be the first thing you would change if you could?

…….

13. Do you work with a multidisciplinary team (paediatric orthopaedic and plastic surgeons, paediatric physiotherapist, paediatrician, psychologist) to help you in the care of paediatric amputees?
   ☐ Yes
   ☐ No
   If yes,
   a. Please specify the team members here:

………

………

   b. Do you think it helps you deliver better care to your patients?
   ☐ Yes ☐ No

   If not,
   a. Why do not you work with a multidisciplinary team?

……

   b. Do you think it would be valuable for you?
   ☐ Yes ☐ No

14. If a psychologist or mental health professional is available, how often do your paediatric patients have an appointment with the psychologist?

……
.

15. What are the main differences between provision for your paediatric versus adult amputees at your centre?

…….

16. How does childhood growth affect your prosthetic provision for paediatric lower limb amputees?

…….

17. What are the main differences between provision for bilateral lower limb loss and unilateral?
   ……

Section 2: Prosthetic Componentry Available

Please take photos of any componentry used in the centre.

Participant ID:

1. What is the main prosthetic technology you offer at your centre?

……

2. Do you receive components from the International Committee of the Red Cross?

☐ Yes ☐ No

   a. If yes, what is your primary method to connect components together?

……….

   b. Do you use welding? ☐ Yes ☐ No

3. Do you sometimes use other technologies from suppliers like Ottobock?

☐ Yes ☐ No

   a. If yes, which components?

……

   b. Would you offer any of these to children?

……

4. Please list all the types of components that can be offered to children in your centre both free and at a charge. Specify which are free of charge?

……….

……….

5. What is the most common type of socket you make for transtibial paediatric amputees? Circle all that apply.
   ☐ Patellar Tendon Bearing Socket ……………….
   ☐ Total Surface Bearing Socket ……………….
   ☐ Other: …………………………………………
6. What is the most common type of suspension you use for transtibial paediatric amputees? Circle all that apply.
   ☐ Supracondylar suspension ……………….
   ☐ Supracondylar suprapatellar suspension ……………….
   ☐ Passive suction (suspension sleeve)……………….
   ☐ Locking suspension system ……………….
   ☐ Other: …………………………………………
7. What is the most common type of liner you use for transtibial paediatric amputees? Circle all that apply.
   ☐ PE-lite liner/foam ……………….
   ☐ Silicone cushion liner with suspension sleeve ……………….
   ☐ Silicone cushion liner without suspension sleeve ……………….
   ☐ Silicone Locking Liner ……………….
   ☐ None ……………….
   ☐ Other: …………………………………………
   Please explain your choices. What are the advantages of the liner you most commonly use?

Participant ID:

………

………

8. What is the most common type of socket you make for knee disarticulation paediatric amputees? Circle all that apply.
   ☐ Push-fit socket ……………….
   ☐ Panel Opening …………….
   ☐ Ischial Bearing ……………….
   ☐ Other: …………………………………………
9. What is the most common type of suspension you use for knee disarticulation paediatric amputees? Circle all that apply.
   ☐ Condylar suspension ……………….
   ☐ Passive suction with one way air valve……………….
   ☐ Silesian belt ……………….
   ☐ Other: …………………………………………
10. What is the most common type of liner you use for knee disarticulation paediatric amputees?
    ☐ PE-lite liner/foam ……………….
    ☐ Silicone cushion liner with suspension sleeve ……………….
    ☐ Silicone cushion liner without suspension sleeve ……………….
    ☐ Silicone Locking Liner ……………….
    ☐ None ……………….
    ☐ Other: …………………………………………
    Please explain your choices. What are the advantages of the liner you most commonly use?

………

………

11. What is the most common type of socket you make for transfemoral paediatric amputees? Circle all that apply.
    ☐ Quadrilateral Socket ……………….
    ☐ Ischial Containment Socket……………….
    ☐ Other: …………………………………………
12. What is the most common type of suspension you use for transfemoral paediatric amputees? Circle all that apply.
    ☐ Passive suction with one way air valve ……………….
    ☐ Active suction ……………….
    ☐ Silesian belt ……………….
    ☐ Locking mechanisms ……………….
    ☐ Magnet suspension ……………….
    ☐ Other: …………………………………………
13. What is the most common type of liner you use for transfemoral paediatric amputees? Circle all that apply.
    ☐ PE-lite liner/foam ……………….

☐ Silicone cushion liner with suspension sleeve ……………….
☐ Silicone cushion liner without suspension sleeve ……………….
☐ Silicone Locking Liner ……………….
☐ None ……………….
☐ Other: …………………………………………
Please explain your choices. What are the advantages of the liner you most commonly use?
………
………

14. What is the most common type of socket you make for hip disarticulation paediatric amputees? Circle all that apply.
☐ None ……………….
☐ Other: …………………………………………
15. What is the most common type of suspension you use for hip disarticulation paediatric amputees? Circle all that apply.
☐ Passive suction with one way air valve……………….
☐ Active suction ……………….
☐ Silesian belt ……………….
☐ Locking mechanisms ……………….
☐ Magnet suspension ……………….
☐ Other: …………………………………………
16. What is the most common type of liner you use for hip disarticulation paediatric amputees? Circle all that apply.
☐ PE-lite liner/foam ……………….
☐ Silicone cushion liner with suspension sleeve ……………….
☐ Silicone cushion liner without suspension sleeve ……………….
☐ Silicone Locking Liner ……………….
☐ None ……………….
☐ Other: …………………………………………
Please explain your choices. What are the advantages of the liner you most commonly use?
………
……

17. What is the most common type of prosthetic knees you use for knee-disarticulation paediatric amputees?
☐ ICRC monocentric paediatric knee ……………….
☐ ICRC monocentric adult knee ……………….
☐ ICRC polycentric adult knee ……………….
☐ Another monocentric passive knee: Specify ………………………… ……………….
☐ Other Polycentric passive Knee: Specify: ……………….
☐Hydraulic Knee: Specify: ……………
☐ Microprocessor Knee: Specify: …………
☐ Other: …………………………………………
☐ None (straight leg): …………………………………………

Please explain your choices. What are the advantages of the knee you most commonly use?
……
……….

18. What is the most common type of prosthetic knee you use for transfemoral paediatric amputees?
☐ ICRC monocentric paediatric knee ……………….
☐ ICRC monocentric adult knee ……………….
☐ ICRC polycentric adult knee ……………….
☐ Another monocentric passive mechanical knee: Specify ………………………… ……………….
☐ Other Polycentric passive mechanical Knee: Specify: ……………….
☐Hydraulic Knee: Specify: ……….
☐ Microprocessor Knee: Specify: ………..
☐ Other: …………………………………………
☐ None(straight leg): …………………………………………
Please explain your choices. What are the advantages of the knee you most commonly use?
……
……….

19. What is the most common type of prosthetic knee you use for hip-disarticulation paediatric amputees?
☐ ICRC monocentric paediatric knee ……………….
☐ ICRC monocentric adult knee ……………….
☐ ICRC polycentric adult knee ……………….
☐ Another monocentric passive mechanical knee: Specify ………………………….
☐ Other Polycentric passive mechanical Knee: Specify: ……………………
☐Hydraulic Knee: Specify: ………..
☐ Microprocessor Knee: Specify: ………………
☐ Other: …………………………………………
☐ None(straight leg): …………………………………………
Please explain your choices. What are the advantages of the knee you most commonly use?
……
……….

20. What is the most common type of adapter/connection mechanism you use for paediatric amputees?
☐ ICRC polypropylene welding ……………….
☐ Modular pyramid system ……………….
☐ Other……………….
☐ Please explain your choices. What are the advantages of the system you most commonly use?
……
……….

21. What is the most common type of prosthetic feet you use for paediatric amputees?
☐ SACH foot sourced from ICRC ……………….

☐SACH foot sourced from another supplier ……………….
☐ ESAR foot ……………….

☐ Other

Please explain your choices. What are the advantages of the feet you most commonly use?

………

………

22. What is the most time-consuming aspect in creating a paediatric prosthetic limb? Does this change for different ages of children?

………

    a. How long does this take?

………

23. What is the main challenge during fitting of the new device? Does this change for different ages of children?

………

………

Section 3.1: Sockets

1. What are the main challenges you experience with paediatric sockets?

………

………

    a. Why did you choose ….. as the main challenge you experience with paediatric sockets?

………

    b. Please rank them in order of importance

☐ Challenges in manufacturing a socket that can last as much as possible
☐ Challenges in fitting
☐ Challenges in use
☐ Challenges in repairing
☐ Challenges in adjusting
☐ Other (please specify) ………………………………………………….

Socket fabrication

2. What do you need to consider when producing a prosthetic socket for a child?

………

3. How long does it take you to make a socket?

……

4. How much time passes from initial casting visit to fitting visit (e.g., 2 weeks, 1 month, few months)?
   ☐ 1 week
   ☐ 2 weeks
   ☐ 4 weeks
   ☐ Few months
   ☐ Other (please specify): …………………………….
5. Do you always consider the child will grow when you make a prosthetic socket?
   ☐ Yes ☐ No
   a. How do you account for growth when making a prosthetic socket?
      ………Circle all that apply.
   ☐ Make the socket bigger and add more inner flexible layers (e.g., 3D printed/EVA foam)
   ☐ Use multiple 5-ply socks
   ☐ Add a pad at the distal end
   ☐ Add removable pads in other parts of the limb
   ☐ Other (please specify): ………………………………….

   b. Out of the techniques you have mentioned, what is your preferred and most used technique to increase socket lifespan? And why?

……

……

……

6. Do you make check sockets? ☐ Yes ☐ No
7. In your opinion, what is the main challenge in paediatric socket fabrication?

……

……

Socket adjustability and replacements

8. What are your techniques to adjust the socket as the child grows to avoid fabricating a new socket every time? Circle all that apply.
   ☐ Thermoforming
   ☐ Adding pads to release pressure from painful areas
   ☐ Grind off material from the socket
   ☐ Change liner/ply socks
   ☐ 3D printed inner flexible socket
   ☐ Other (please specify): ………………………………….
9. How do you determine when an adjustment is needed (e.g., gait analysis, anthropometric measurements)?

……

Participant ID:

……….

……….

10. Does the child need to come to the centre every time an adjustment is required?
    ☐ Yes ☐ No

11. Please circle the most correct statement below. The **adjustments** are:

    ☐ Mostly circumferentially

    ☐ Equally circumferentially and longitudinally

    ☐ Mostly longitudinally

12. How often is an adjustment required for:
    a. Children aged 3-5: ……………………… (Specify measure of unity)
    b. Children aged 6-8: ……………………… (Specify measure of unity)
    c. Children aged 9-12: ……………………… (Specify measure of unity)
    d. Children aged 13-15: ……………………… (Specify measure of unity)
    e. Children aged 16-18: …………………… (Specify measure of unity)

13. Please circle the most correct statement below. The prosthetic **replacements** are due to:

    ☐ Mostly circumferential growth

    ☐ Equally circumferential and longitudinal growth

    ☐ Mostly longitudinal growth

14. How often do you completely replace the socket for:

    a. Children aged 3-5: ……………………… (Specify measure of unity)
    b. Children aged 6-8: ……………………… (Specify measure of unity)
    c. Children aged 9-12: ……………………… (Specify measure of unity)
    d. Children aged 13-15: ……………………… (Specify measure of unity)
    e. Children aged 16-18: ……………………… (Specify measure of unity)

15. What type of socket material do you prefer the most and why?
    ☐ Polypropylene paediatric socket because …………………………………………………………………….

    ……….

    ☐ No preference because ……………………………………………………………………………………

    …….

    ☐ Carbon/glass fibre paediatric socket because ……………………………………………………………

    …….

16. When a child requires a new socket, do you reuse any of the other componentry such as the knee? ☐ Yes ☐ No
    Please circle all the components that you reuse.
    ☐ Knee
    ☐ Pylon, adaptors, and other connecting parts
    ☐ Foot

17. What do you do when a child comes back and there is a leg-length discrepancy between the prosthetic side and intact side? How do you increase the length of the prosthesis?

……

18. How do you reuse the components?

……

    a. Is reusing the components time consuming?

……

    b. How many times can you reuse the same component?

……

19. What is the main complaint you receive from children about their socket?

……

20. What future development is required to make paediatric sockets more appropriate to the child's needs and yours (from material to fabrication to fitting and replacement)? What would you change to improve adjustability?

……

……

Section 3.2: Prosthetic knee

1. Do you offer prosthetic knees to children? ☐ Yes ☐ No
    a. At what age do you offer prosthetic knees? Please specify by type:
    ……
        i. Why this age?

……

……

2. Based on what measure do you prescribe a knee joint? E.g., on a child's age, or an anthropometric measure such as height or weight, or a gait developmental milestone?

……

3. What are the main challenges with the prosthetic knees you can offer the children?

……

4. What are the current limiting factors for providing a child with a knee joint? Please breakdown by age group.

Participant ID:

……

……

5. Do you usually provide a child with a locked or unlocked knee?
   ☐ Locked ☐ Unlocked
   a. Why?
      ……
   b. At what age or stage in rehabilitation would you encourage the knee to be unlocked?
      ……
   c. What are the limiting factors on unlocking the knee joint?
      ……
   d. Is it important to have a locking mechanism in the knee?
      ……
      i. Why?
         ………
6. How do you connect the knee to the socket?

……

7. What are the challenges in manufacturing and fitting the prosthetic knees you use?

……

8. What are the challenges with alignment for each knee you currently have access to?

……

9. How often does a child require a new prosthetic knee? Please specify for each knee type available for prescription:

……

   a. Does this change for different age groups?
      ……
   b. Why would they usually need a new knee?
      ……
   c. Would you ever reuse the same prosthetic knee with a different prosthesis?
      Yes: ☐ No: ☐
      i. When?
         ………
      ii. Why?
         ………
      iii. How many times?
         ………
10. How does the child's growth during childhood affect their use of a prosthetic knee joint?

……

11. When do you move children onto adult knees?

……

Participant ID:

a. Why do you move them from children to adult knee?

……

12. What do you think about the stability of the prosthetic knees you can prescribe?

……

13. What is your opinion on the weight of the knees you can offer children?

……

14. What is your opinion on the size of the knees you can offer children?

……

15. What is your opinion on the appearance of the knees you can offer children?

……

16. What is your opinion on swing phase control or extension assist, do you think this is an important feature for an ideal knee?

……

a. What are the challenges with current swing phase control/ extension assist mechanisms?

……

17. What is the main complaint you receive from children regarding their prosthetic knee?

………

a. Does this change with age?

18. Do you receive any complaints about the sound the prosthetic makes during walking?

……

19. Where does each type of knee you use usually wear out or break?

……

a. How often are repairs required?

……

b. Is it easy to repair the knees? If so, what repairs do you usually make for each knee type?

………

20. What future development is needed to make paediatric knee componentry more appropriate to the child's needs? What would you suggest for change?

………

………

………

………

21. Would a low-cost appropriate polycentric knee joint for children be a useful innovation?

………

Section 3.3 Prosthetic Foot

1. What are the main challenges with the prosthetic feet you provide to children?

………

2. Where do the feet usually wear out?

………

3. What is the main complaint children have about their prosthetic feet?

………

4. How does growth affect prosthetic foot provision?

………

Section 4: Assistive technology

1. Do you provide other mobility aids instead of a prosthetic limb? ☐ Yes ☐ No
   a. Which aids?
   ……
2. When do you provide mobility aids other than a prosthetic limb? Tick all that apply.
   ☐ post-surgery for primary patients
   ☐ Residual limb conditions like blisters that prevent use of prosthesis
   ☐ Bone overgrowth that prevents use of prosthesis
   ☐ Additional walking aid for patients who need extra support
   ☐ Other (please specify): …………………………………………………

Participant ID:

Section 5: Sourcing Componentry

1. How does supply chain work in the facility where you work (e.g., tender for calls or placing orders as required)?

   ………

2. Where do you source the componentry from (which country and which supplier)?
   a. Country/countries (Please specify all): ……………………………………………………………………

      ……

   b. Supplier/suppliers (Please specify all): ……………………………………………………………………

      ……

3. How frequently do you order components?

   ☐ Whenever I need them

   ☐ Every month

   ☐ Every three months

   ☐ Every six months

   ☐ Once a year

   ☐ Other (please specify): ………………………………………………………………………

4. How long do you usually wait before the supplies arrive to the centre (e.g., few days, few weeks, or few months)?

……

5. Is the waiting time a limiting factor in your prosthetic service provision? E.g., you wait an extra month before being able to deliver the prosthetic leg.

……

6. How frequently does the supplier change? ……………………………………………………………
   a. Do you know why? ……………………………………………………………………………………………

7. Who chooses the componentry and the supplier? Please tick the right box.

   ☐ You

   ☐ The hospital/centre administration

   ☐ Someone else such as the government (Please specify below)

……

Participant ID:

Section 6: Residual limb health:

1. What are the main challenges you face for residual limb health in children? What is the biggest challenge of those?

☐ Bone overgrowth/bone spurs ……………….

☐ Blisters ……………….

☐ Skin redness……………….

☐ Abrasions……………….

☐ Other: ……………………………………….

Please list all the other problems related to residual limb health.

………

………

………

2. What are the causes of the problems in the residual limb listed above? Please circle all that apply and rank them in order of importance.

   ☐ Growth

   ☐ Socket

   ☐ High-activity level

   ☐ Other: ……………………………………….

3. Do you see cases of bony overgrowth/bone spurs? ☐ Yes ☐ No
   a. How often?

………

4. What age is bony overgrowth most likely to occur?

   ☐ <10yrs

   ☐ 10-15 yrs

   ☐ above 15yrs

5. Do different amputation levels have increased likelihood of displaying bone overgrowth? Which ones are the most?

   ☐ Partial Foot Amputation

   ☐ Ankle Disarticulation

   ☐ Transtibial/Below Knee

   ☐ Knee Disarticulation

   ☐ Transfemoral/Above Knee

   ☐ Hip Disarticulation

   a. What are the typical symptoms?

………

   b. What is the standard procedure once you have identified overgrowth?

Participant ID:

……

……

c. What are your techniques to deal with overgrowth during prosthetic fitting?
……….

d. Does the child have to stop wearing a prosthetic, if so, do you provide another mobility aid?
……
…………………………

e. Do you refer or encourage the child to seek out any other treatment such as revision surgery? ☐ Yes ☐ No

6. Do you have knowledge of any revision surgeries that occur? ☐ Yes ☐ No

a. Do you track patients between different surgeries?

……

……

……

Section 7: Gait Training

1. Do you have a set protocol for gait training after prosthetic provision, please describe?

……

……

2. How do you analyse the progress of the child during gait training?

……

……

3. What facilities do you have at the centre for gait training? Circle all that apply.
☐ Parallel bars
☐ Ramps
☐ Stairs
☐ Uneven ground
☐ Obstacles
☐ Other (please specify): ……………………………………………………….

Section 8: Policy and Data Storage:

1. What is your protocol for data storage? Do you keep a case file on each user?
☐ Yes ☐ No

a. If yes, is this on paper or online?
☐ Paper ☐ Online

b. Do you update these files each time a child attends the centre?
☐ Yes ☐ No

c. What information do you store on file?

……

d. Do you store the anthropometric data of the child including the residuum measurements?

☐ Yes ☐ No

e. Do you keep a record of each prosthetic componentry fitted and when?

☐ Yes ☐ No

f. Do you keep a record of residual limb health and the occurrence of any revision surgeries?

☐ Yes ☐ No

g. Can we access and make copies of these files to analyse for research purposes?

☐ Yes ☐ No

h. If a child leaves the centre, how long do you keep the case file for?

2. Are you in contact with the surgical team conducting the amputation surgeries?

☐ Yes ☐ No

3. Do you have access to any medical imaging reports the child has received such as X-Ray?

☐ Yes ☐ No

b. If yes, do you use these to inform prescription?

4. Do you have X-Ray imaging facilities? ☐ Yes ☐ No

5. Do you have a 3D scanner? ☐ Yes ☐ No

Section 9: Continuing Care

1. Do you follow up with families to check on the child or remind them to attend an appointment or do you wait until they return? ☐ Yes ☐ No

If you follow up with families,

a. Why is that necessary? Aren't the families coming as often as they should?

……

b. How do you follow up? Phone call, email etc?

……

c. How often?

……

If you do not follow up with families,

d. Why is it not necessary? Do they come to the centre when they have to?

……

2. What services are given free of charge at your centre and for what do clients have to pay?

a.	Prosthetic Componentry	Free:	☐	Client Pays:	☐
b.	Prosthetic Fitting	Free:	☐	Client Pays:	☐
c.	Accommodation:	Free:	☐	Client Pays:	☐
d.	Travel to Centre	Free:	☐	Client Pays:	☐
e.	Food:	Free:	☐	Client Pays:	☐
f.	Other: …………………………	Free:	☐	Client Pays:	☐

Participant ID:

3. If services are given free of charge, who covers the cost?

……….

4. Do you organise out-reach visits to reach out to paediatric amputees and their family who unaware of the prosthetic service you provide, and they require?

……….

5. Do you think the majority of the lower-limb paediatric amputees in your region have access to prosthetic care? Yes: ☐ No: ☐
   a. **If not**, what are the main reasons? Which would you say has the largest impact? ………
      - ☐ Financial problems ……
      - ☐ Distance and time needed to travel to the centre ……
      - ☐ Lack of awareness ………
      - ☐ Stigma
      - ☐ Other (please specify reason) ……………………….
