# Supplementary material for: Listening to children with lower limb loss: Rationale, design, and protocol for delivery of a novel globally applicable research toolkit—Prosthetic user needs, quality of life, pain, and physical function
Source: PLoS One. 2024 Oct 31;19(10):e0310848. doi: 10.1371/journal.pone.0310848 (PMC11527159; doi:10.1371/journal.pone.0310848)
Supplement: S4 File — (PDF) [file pone.0310848.s004.pdf]

PARTICIPANT ID NUMBER: ………………………

Questionnaire for children above 10 years old

Interviewer should talk a bit with the children and gain their trust before starting the questionnaire. This is particularly important as the children can understand the questions that will be asked, but it is possible they do not understand the purpose of the questionnaire. It is highly recommended to spend time with them/talk to them until they feel at ease and before starting the questionnaire.

Please follow the script sections written with "" marks throughout and continue chatting and getting the child to be relaxed.

If the child keeps talking during the interview, let them, and take notes. This questionnaire is meant to contain everything you will need to ask appropriate questions but if the child is continuing to talk and be open feel free to add in a few appropriate questions to keep the conversation flowing. Do not stray too far off topic.

If the child shrugs or displays answers through body language, please note down.

Please make it clear to the parent/guardian present that they will have their own questionnaire and time to give their opinion so they should not interrupt their child or give their opinions at this stage.

Tick the correct answer for yes/no questions.

If at any time the child is too tired or too distressed to continue, stop the interview.

Do make sure the child is not an orphan. Otherwise, avoid family related questions.

All cards and drawings used during the interview should be kept with the completed documents and stored together.

Start the audio recording if consent was given.

For children up to 14 years old:

"Thank you for letting us chat to you today. I am from a group of people working to help understand how we can make better prosthetic legs for children across the world. We would love your help to do this. Would you be able to teach us how you feel and what you think about the leg you have been given? Can we do some drawing and games while asking you a few questions?"

Wait for them to say yes.

"If you get tired or want to stop at any point just let me or your 'parent/sister/whoever is there' know or just give me a thumbs down *(mimic)* and we will take a break. Okay, let's get started with a few drawings, I'm going to join in too!"

For children above 14 years old:

"Thank you for letting us chat to you today. I am from a group of people working to help understand how we can make better prosthetic legs for children across the world. We would love your help to do this. Would you be able to teach us how you feel and what you think about the leg you have been given? Can ask you a few questions?"

Wait for them to say yes.

"If you get tired or want to stop at any point just let me or your 'parent/sister/whoever is there' know or just give me a thumbs down *(mimic)* and we will take a break. Okay, let's get started!"

PARTICIPANT ID NUMBER: ………………………

Location of interview: …………………………………
Date: Day: ……… Month: ………. Year:…………

Check the audio is recording.

For children up to 14 years old, start by asking them to join you to draw themselves and their family. Give them blank paper and coloured pencils and join in with the drawings to start to make them feel at ease and remove the power dynamic. If they do not want to draw, give them a colouring in book and spend 5 minutes colouring in with them.

Section 1: Amputation experience and daily life challenges:

"Now we would like to ask you a few questions and I am just going to make some notes so I can remember what you said if that's okay?"

1. Why did you come to the clinic today?
………
2. What did you do during the day yesterday?
………
3. What would a good/fun/happy day look like for you?
………
4. Do you want to tell me about your prosthetic leg, how it works, what are the different parts? ***Make them feel as they are the expert of the prosthesis.***
………
5. Do you like it? Does it give you any problems?
………

For children up to 14 years old, ask them to draw their dream prosthetic leg and explain it.

6. What clothes do you like to wear? ***Compliment them on their choices.***
………
    a. Can you explain why?
    ……………………………………………………………………………………………
7. ***For those who said a long dress/skirt/religious dress:*** Can you easily walk when wearing this?
………

Section 2: Mobility and Prosthesis Use:

1. Do you wear your prosthetic leg every day? Yes: ☐ No: ☐
    a. ***If no,*** when do you wear your limb?
    ……………………………………………………………………………………
2. Do you wear your prosthetic leg all day? Yes: ☐ No: ☐
    a. ***If no,*** when do you usually take it off?
    ……………………………………………………………………………………
3. Where do you wear your prosthetic leg? (Home, school etc.)
……………………………………………………………………………………………
4. Do you wear it at home? Yes: ☐ No: ☐

PARTICIPANT ID NUMBER: ………………………

5. Would you rather have a walking aid or a prosthesis? ***Get them to pick a card then note a tick mark on the card chosen.***

a. Why? ……………………………………………………………………………………………………

Walking Aid ☐

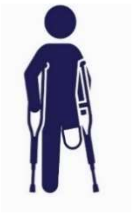

Prosthesis ☐

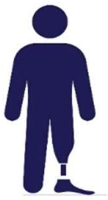

“For this next activity we would like you to split this set of cards into two groups, one group is the activities you can do, and the other group is the activities you cannot do. If you do not understand a picture, let me know and I will try and explain.”

Please then place a tick on those they are comfortable with. Please also note down anything they say about each activity. Exchange cards according to typical cultural activites for environment of use if necessary.

**Walking without cane or crutches ☐**	**Going up the stairs ☐**	**Going down the stairs ☐**
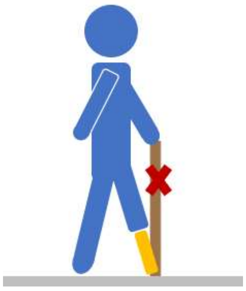	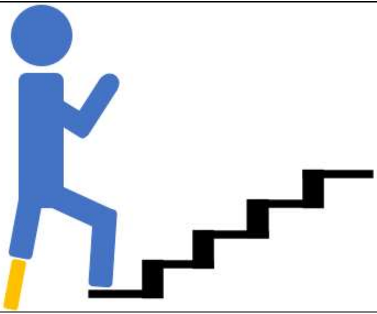	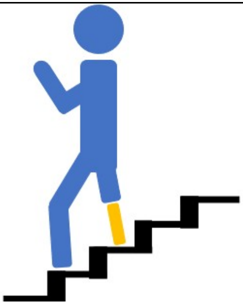
**Walking fast ☐**	**Walking on field/uneven terrain ☐**	**Putting my shoes on alone ☐**
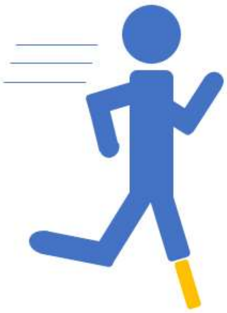	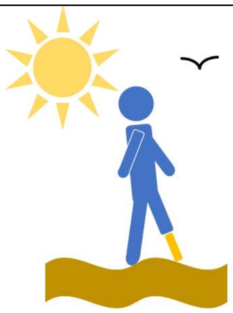	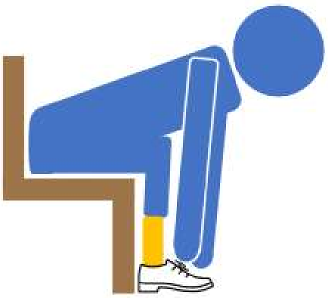
**Walking uphill ☐**	**Walking downhill ☐**	**Squatting ☐**
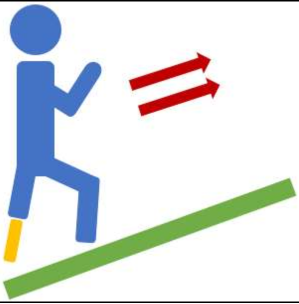	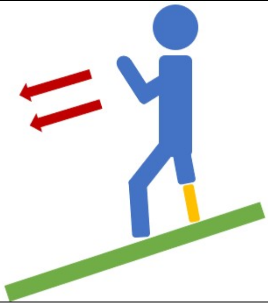	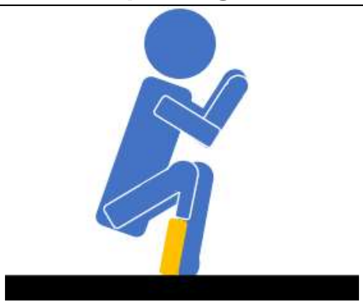

PARTICIPANT ID NUMBER: ……………………….

Sitting with legs crossed ☐	Kneeling ☐	Jumping ☐
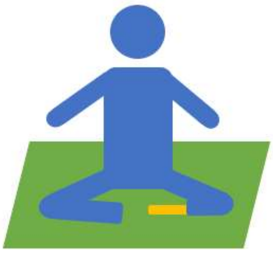	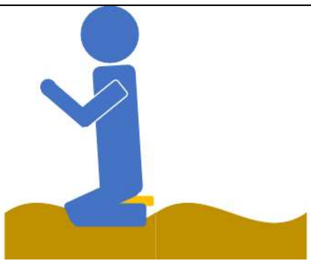	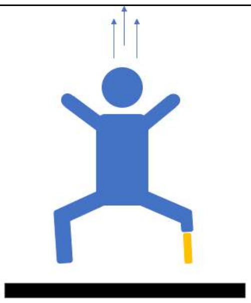

Note down any opinions on the activities above:

………

………

………

6. Are there any other activities that you want or like to do? ***If they do not say anything give examples such as running or jumping and see if they give any more insight.***

   ………

   ………

Section 3: Sport and Leisure

1. ***If a high resourced environment, else skip to 2:*** Do you have more than one prosthetic leg?

……

   a. If yes, which is your favourite?

……

      i. Tell me about when you use each one?

……

   b. If no, can you do everything you want to with this leg?

……

2. What is your favourite sport?

……

   a. Do you watch it or play it as well?

……

3. Do you play sport at school?

……

PARTICIPANT ID NUMBER: ………………………

Section 4: Prosthetic Componentry

1. Which one of these four cards do you prefer? Which one would you like to have the most? ***Current cards are for low resourced environment, exchange cards according to environment of use, i.e. ensure more modular componentry options for high resourced environment. Current cards are for transtibial amputation level, exchange cards for other levels if necessary (see full toolkit).***
    a. Why? ……………………………………………………………………………………………

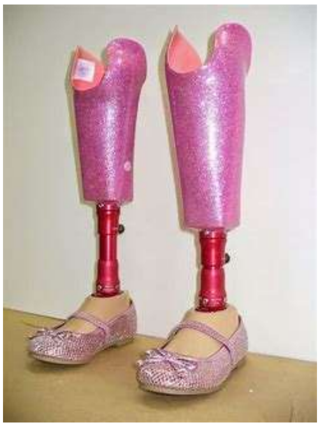

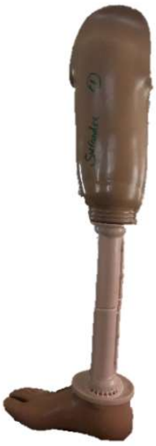

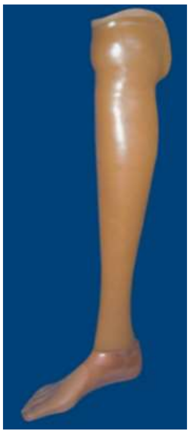

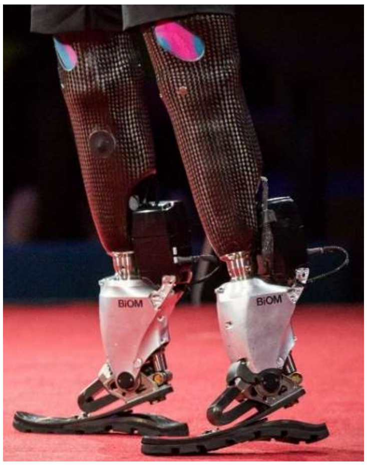

Sparkling Legs ☐ **Prosthetic without Cover ☐** **Prosthetic with Cover ☐** **Bionic limbs ☐**

Section 4.1: Socket Componentry

"We are now going to discuss how you feel about your prosthetic socket."

Interviewer, point to an example socket.

1. Do you like your socket? Yes: ☐ No: ☐
    a. Tell me more about it. Is it comfortable?
    ……………………………………………………………………………………………………
    b. Does your socket cause you any challenges? (e.g. Does it cause you to go to the prosthetic clinic often? Does it cause you to stop using your prosthetic leg often?)
    ……………………………………………………………………………………………………
2. What would you change in your socket?
……………………………………………………………………………………………………
3. Which one of these two cards do you like the most? ***Exchange cards according to amputation level if necessary.***
    a. Why?
    ……………………………………………………………………………………………………
    b. Would they wear the adjustable one if it were more comfortable?
    ……………………………………………………………………………………………………

Typical socket

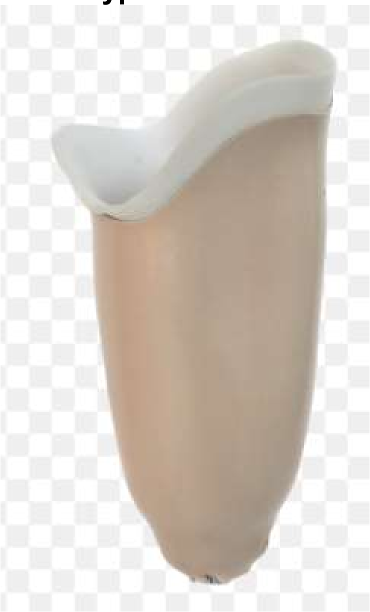

Adjustable socket

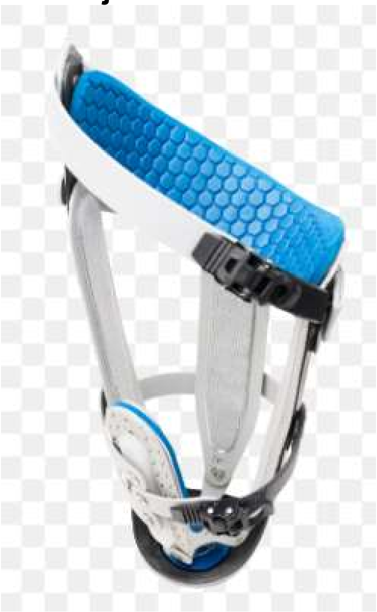

4. Does your limb get sweaty inside your socket? **"I *know that I can get sweaty in the hot weather too!"***
   ………
   a. When does this happen?
      ………………………………………………………………………………………………………

Section 4.2: Prosthetic Knee

Only complete this section if the child is a transfemoral or knee disarticulation amputee and therefore only if the child would ever require a prosthetic knee. Select the appropriate section of questions for the child based on whether they have an unlocked/locked or no knee joint.

If child has a knee but it is locked straight or no knee at all:

1. Do you find your straight leg helpful? Yes: ☐ No: ☐
   a. Why? ……………………………………………………………………………………………
2. Do you want to change anything about your straight leg?
   ……………………………………………………………………………………………………
3. Would you like to be able to bend your leg at the knee? ***Mimic knee bending***
   Yes: ☐ No: ☐
   a. Can you explain why?
      ………………………………………………………………………………………………
4. Do you feel stable?
   ……………………………………………………………………………………………………
5. Are you able to walk as fast as you want? Yes: ☐ No: ☐
   ……………………………………………………………………………………………………

If child has an unlocked articulating knee that can swing freely:

1. Do you find it helpful to have your knee bending like this? ***Interviewer mimic bending knee***
   Yes: ☐ No: ☐
   a. Why? ………………………………………………………………………………………

2. Do you want to change anything about how your knee works?
   ……
3. Would you like to be able to keep your leg straight? Yes: ☐ No: ☐
   a. Can you explain why?
      ……………………………………………………………………………………………………
4. Can you lock your knee yourself to make your leg stay straight? ***Interviewer mimic straight leg***
   Yes: ☐ No: ☐
   a. Or does someone else lock your knee or make it stay straight?
      Yes: ☐ Who? .... No: ☐
      i. Can you explain why?
         ……………………………………………………………………………………
5. Has the knee ever got caught in your clothes?
   ………
6. Do you feel in control of your knee? Do you feel stable?
   ………
   ………
7. Does your knee make any noises? Yes: ☐ No: ☐
   a. ***If yes***, when? What do you think of the sound?
      ………
8. Are you able to walk as fast as you want? Yes: ☐ No: ☐
   ………
9. Do you like the way your knee looks? Yes: ☐ No: ☐
   a. Do you like the size?
      ……

Section 4.3: Foot

"We are now going to discuss how you feel about your prosthetic foot" ***Discuss blade instead of foot if required in high resourced environment.***

Interviewer, point to an example foot.

1. Do you like your foot? ☐ Yes ☐ No
   b. Tell me more about it. Is it comfortable?

……

   c. Does your foot cause you any challenges?

……

2. What would you change in your foot?

……

If in high resourced environment and child is using blade, check whether child also uses typical foot as well as blade. If yes:

3. When do you use each type and for what do you use it?
……..

PARTICIPANT ID NUMBER: ………………………

The next activity is a ranking activity. Exchange cards according to typical cultural activites for environment of use if necessary.

"Can you please rank these 10 cards in order of the things that you want the most. The things you want to be able to do or have in your life the most you put at the top and the things you are not interested in you put at the bottom. This is not about what you can or cannot do, but what you want to do. For example, do you want to walk faster **more** than you want to kneel?" ***Please take the time to make sure they understand it is about future desire not current capability.***

Write the ranking number on the cards after the child has finished.

Walk faster/Run	Be less tired by the end of the day	Kneel
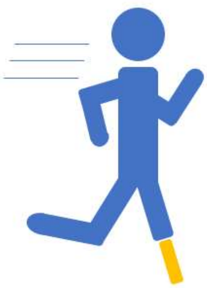	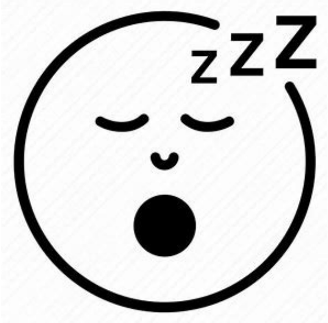	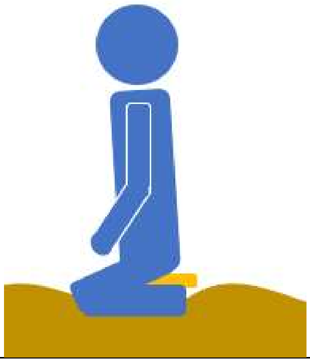
**Visit the clinic less**	**Feel more stable when standing and walking on your leg**	**Less pain in my residual limb**
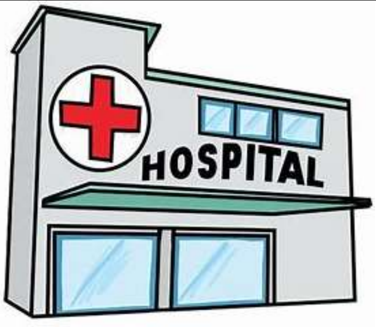	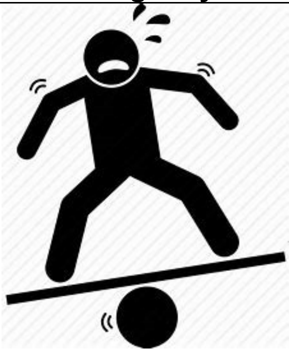	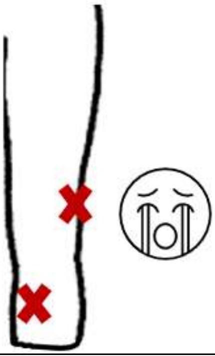
**Walk more quietly**	**Sit with my legs crossed**	**Squat**
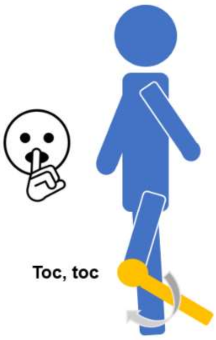	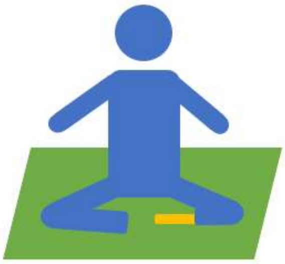	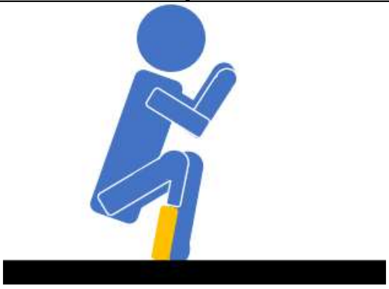
**Walking without any help**		

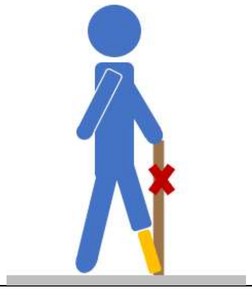		

8. Are there any other important things you would like to change or improve in your daily life?
……

Section 5: Physical Impact

1. At the end of the day, where in your body do you get tired first?
   ………..
2. How do you deal with any pain you feel? When you feel pain do you take off your prosthetic leg?
   ………
3. Do you like going to sleep at night?
   ……
   a. Can you explain why? ………………………………………………………………………………
4. Do you wake up in the night?
   ………
   a. What happens when you wake up?
      ……………………………………………………………………………………………………
5. Finally, do you have questions for me?

PARTICIPANT ID NUMBER: ....

Draw yourself.

PARTICIPANT ID NUMBER: ....

Draw your family.

PARTIPANT ID NUMBER: ………………………

Draw your dream prosthetic leg.
