# Supplementary material for: Listening to children with lower limb loss: Rationale, design, and protocol for delivery of a novel globally applicable research toolkit—Prosthetic user needs, quality of life, pain, and physical function
Source: PLoS One. 2024 Oct 31;19(10):e0310848. doi: 10.1371/journal.pone.0310848 (PMC11527159; doi:10.1371/journal.pone.0310848)
Supplement: S5 File — (PDF) [file pone.0310848.s005.pdf]

PARTICIPANT ID NUMBER: ………………………

Questionnaire for children below 10

Interviewer should play and talk with the children to gain their trust before starting the questionnaire, especially if they do not know the interviewer. Children usually require some time before opening up. It is recommended to play and talk to them before starting the questionnaire. It is also important to explain to them why we are doing this.

Please follow the script sections written with "" marks throughout and continue chatting and getting the child to be relaxed.

If the child keeps talking during the interview, let them, and take notes. This questionnaire is meant to contain everything you will need to ask appropriate questions but if the child is continuing to talk and be open feel free to add in a few appropriate questions to keep the conversation flowing. Do not stray too far off topic.

If the child shrugs or displays answers through body language, please note down.

Please make it clear to the parent/guardian present that they will have their own questionnaire and time to give their opinion so they should not interrupt their child or give their opinions at this stage.

Tick the correct answer for yes/no questions.

If at any time you feel the child is too tired or too distressed to continue, pause the interview, check the child is happy to continue. If not, stop the interview.

Do make sure the child is not an orphan. Otherwise, avoid family related questions.

All cards and drawings used during the interview should be kept with the completed documents and stored together.

Be aware of maturity level and be prepared to explain question in multiple ways until the child understands.

Start the audio and video recording if consent was given.

"Thank you for letting us talk to you today. I am from a group of people working to understand how we can build better prosthetic legs for children across the world. We would love you to help us. Would you be able to teach us how you feel and what you think about the leg you have been given? Can we do some drawing and games while asking you a few questions?"

Wait for them to say yes.

"If you get tired or want to stop at any point just let me or your 'parent/sister/whoever is there' know or just give me a thumbs down *(mimic)* and we will take a break. Okay, let's get started with a few drawings, I'm going to join in too!"

PARTIAL ID NUMBER: ………………………

__Check the audio is recording.__

Location of Interview: ……………………………………………………………………………

Date of Interview: Day: ……….. Month: ………. Year: ……….

Start by asking them to draw themselves and their family. Give them blank paper and coloured pencils and make sure you join in with the drawing.

Section 1: Social

"Now we would like to ask you a few questions and I am just going to make some notes so I can remember what you said if that's okay?"

1. Do you go to school? Yes: ☐ No: ☐
   a. ***If yes***, do you like school?
   ………………………………………………………………………………………………
   b. ***If no,*** what do you do during the day?
   ………………………………………………………………………………………………
2. Do you like to walk a lot? Yes: ☐ No: ☐
   a. ***If not***, why not?
   ………………………………………………………………………………………………
3. Which games do you play? Tell me more about the games you like. Who do you play with?
   ………………………………………………………………………………………………

Section 2: Amputation background

1. Do you have a prosthetic leg? Yes: ☐ No: ☐

If they use a prosthetic,

2. Do you want to tell me about your prosthetic leg (***point to it***): How does it work? (***Make them feel like they are the expert, if the child is not really responding then ask them to draw their prosthetic leg and describe it to you.***
   ………………………………………………………………………………………………
   ………………………………………………………………………………………………
3. Do you like it? Yes: ☐ No: ☐
   a. Can you explain why?
   ………………………………………………………………………………………………
   ………………………………………………………………………………………………
4. Can you draw me your dream prosthetic leg? ***Give them paper and coloured pencils. Compliment drawing.***

5. Can you show me your drawing/explain?
   ………………………………………………………………………………………………

If they do not have a prosthetic limb, ask them to tell you about any mobility aids they use. ………………………………………………………………………………………………

PARTICIPANT ID NUMBER: ………………………

Section 3: Mobility

6. Can you pick your favourite image from these two cards?
   Interviewer, apply a tick to the chosen card.
   a. Why did you pick that one?

……

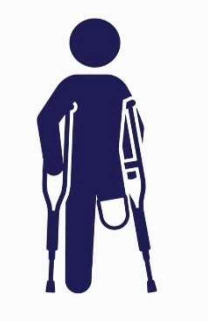

1

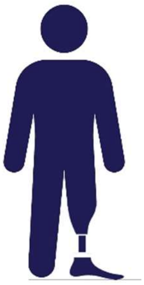

2

7. ***Say "my feet get sore after walking all day."*** Does your leg ***(point to residuum)*** ever get sore:
   a. When you sit down ***(mimic)***? ☐ Yes ☐ No
   b. When you stand up ***(mimic)***? ☐ Yes ☐ No
   c. When you walk ***(mimic)***? ☐ Yes ☐ No

Section 4: Componentry

Only complete this section if the child uses a prosthetic. Current cards are for low resourced environment, exchange cards according to environment of use, i.e. ensure more modular componentry options for high resourced environment. Current cards are for transtibial amputation level, exchange cards for other levels if necessary (see full toolkit).

8. Which one of these legs do you like the most? ***Interviewer, note down on back of card which one was chosen.***

Prosthetic without cosmetic cover

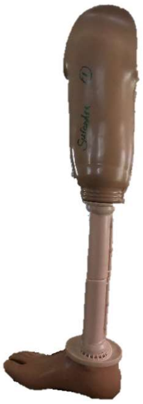

Prosthetic with cosmetic cover

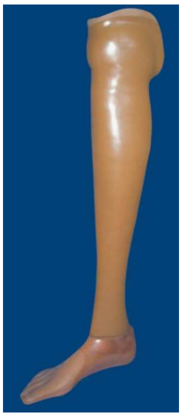

Sparkling legs

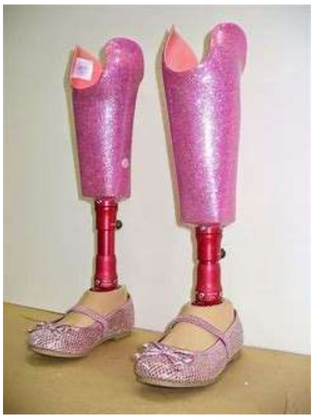

Bionic legs

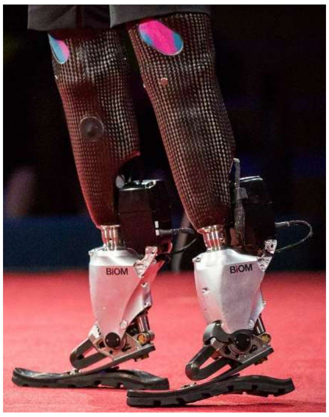

a. Why did you pick that one?

………

9. Can you pick the 3 things you want to do the most? You can only pick 3. Can you tell me why you want these activities the most? ***Exchange cards according to typical cultural activites for environment of use if necessary.***
   ……………………………………………………………………………………………………
   ……………………………………………………………………………………………………

**Walk faster**	**Sit with your legs crossed**	**Squat**
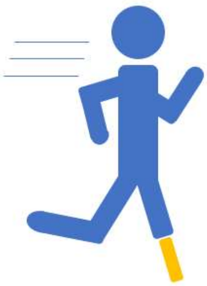	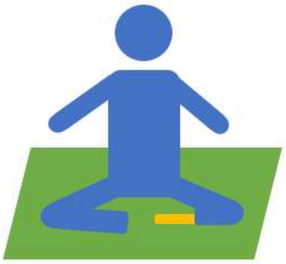	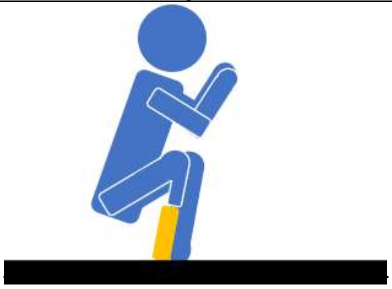
**Visit the clinic less**	**Walking without any help**	**Less pain in my residual limb**
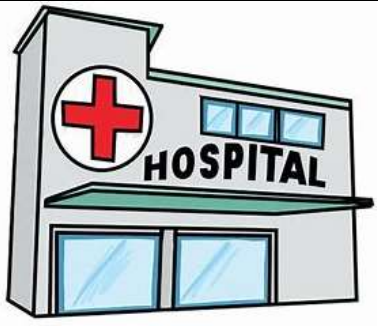	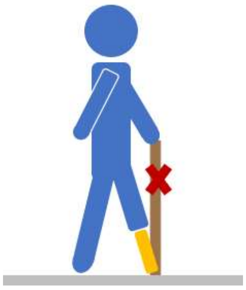	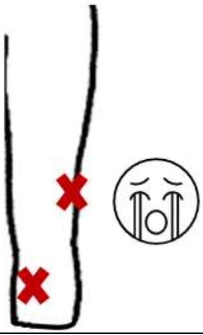
**Kneeling**		
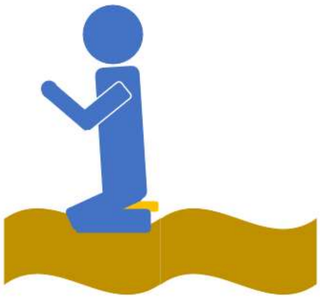		

10. ***If transfemoral or knee-disarticulation,*** what do you think about having a prosthetic knee joint? Can you tell me if it helps you or how it makes you feel?
    ……………………………………………………………………………………………………
11. Do you like to play sports?
    ……………………………………………………………………………………………………
    a. ***If high resourced environment,*** do you have any other prosthetic legs for your sport?
       ………………………………………………………………………………………

PARTICIPANT ID NUMBER: ……………………….

12. Is there anything else you want to change about your prosthetic leg? Or anything else you want to tell me about?

    ……

13. Do you have any questions for me?

    ……

Draw yourself.

PARTICIPANT ID NUMBER: ....

Draw your family.

PARTICIPANT ID NUMBER: ……………………….

Draw your dream prosthetic leg.
